# Two FtsH Proteases Contribute to Fitness and Adaptation of *Pseudomonas aeruginosa* Clone C Strains

**DOI:** 10.3389/fmicb.2019.01372

**Published:** 2019-07-09

**Authors:** Shady Mansour Kamal, Morten Levin Rybtke, Manfred Nimtz, Stefanie Sperlein, Christian Giske, Janja Trček, Julien Deschamps, Romain Briandet, Luciana Dini, Lothar Jänsch, Tim Tolker-Nielsen, Changhan Lee, Ute Römling

**Affiliations:** ^1^Department of Microbiology, Tumor and Cell Biology, Karolinska Institutet, Stockholm, Sweden; ^2^Department of Microbiology and Immunology, Faculty of Pharmaceutical Sciences & Pharmaceutical Industries, Future University in Egypt, New Cairo, Egypt; ^3^Costerton Biofilm Center, Department of Immunology and Microbiology, Faculty of Health and Medical Sciences, University of Copenhagen, Copenhagen, Denmark; ^4^Department of Cellular Proteomics, Helmholtz Centre for Infection Research, Braunschweig, Germany; ^5^Division of Clinical Microbiology, Department of Laboratory Medicine, Karolinska Institutet, Stockholm, Sweden; ^6^Department of Biology, Faculty of Natural Sciences and Mathematics, University of Maribor, Maribor, Slovenia; ^7^Micalis Institute, INRA, AgroParisTech, Université Paris-Saclay, Jouy-en-Josas, France; ^8^Department of Biological and Environmental Sciences and Technologies (DiSTeBA), University of Salento, Lecce, Italy

**Keywords:** *Pseudomonas aeruginosa*, clone C strains, FtsH protease, heat shock factor RpoH, phenazine, secondary metabolite, virulence, autolysis

## Abstract

*Pseudomonas aeruginosa* is an environmental bacterium and a nosocomial pathogen with clone C one of the most prevalent clonal groups. The *P. aeruginosa* clone C specific genomic island PACGI-1 harbors a xenolog of *ftsH* encoding a functionally diverse membrane-spanning ATP-dependent metalloprotease on the core genome. In the aquatic isolate *P. aeruginosa* SG17M, the core genome copy *ftsH1* significantly affects growth and dominantly mediates a broad range of phenotypes, such as secretion of secondary metabolites, swimming and twitching motility and resistance to aminoglycosides, while the PACGI-1 xenolog *ftsH2* backs up the phenotypes in the *ftsH1* mutant background. The two proteins, with conserved motifs for disaggregase and protease activity present in FtsH1 and FtsH2, have the ability to form homo- and hetero-oligomers with *ftsH2* distinctively expressed in the late stationary phase of growth. However, mainly FtsH1 degrades a major substrate, the heat shock transcription factor RpoH. Pull-down experiments with substrate trap-variants inactive in proteolytic activity indicate both FtsH1 and FtsH2 to interact with the inhibitory protein HflC, while the phenazine biosynthesis protein PhzC was identified as a substrate of FtsH1. In summary, as an exception in *P. aeruginosa*, clone C harbors two copies of the *ftsH* metallo-protease, which cumulatively are required for the expression of a diversity of phenotypes.

## Introduction

*Pseudomonas aeruginosa* is a gram-negative opportunistic pathogen causing a broad spectrum of nosocomial infections in individuals with local or systemic immune system deficiency (Lyczak et al., 2000; Kerr and Snelling, 2009; Parkins et al., 2018). Besides its clinical impact, *P. aeruginosa* inhabits environmental niches like soil, water and plants (Goldberg, 2000; Wiehlmann et al., 2015). *P. aeruginosa* clone C strains are a prevalent group of closely related *P. aeruginosa* strains present in natural and clinical habitats (Römling et al., 1994, 2005; De Soyza et al., 2013; Parkins et al., 2018). Clone C strains are characterized by strain-specific genomic islands (Larbig et al., 2002; Kung et al., 2010; Fischer et al., 2016) and a common 102 kbp plasmid (Klockgether et al., 2008; Kung et al., 2010). Features on the core genome, the accessory genome or a combination of both may lead to the capability of clone C strains to colonize different niches (Lee et al., 2015, 2018).

Comparative genome analysis revealed acquisition of the clone C specific genomic island PACGI-1 in the aquatic isolate SG17M and commonly also in other clone C strains (Lee et al., 2015, 2016). The right border of PACGI-1 consists of a gene cluster mainly dedicated to protein homeostasis named the transmissible locus of protein quality control-1, TLPQC-1 (Lee et al., 2016). A fundamental principle of many gene products encoded on PACGI-1/TLPQC-1, namely being xenologs of core genome gene products, has been observed previously for other genomic islands in *P. aeruginosa* (Essar et al., 1990; Liang et al., 2001). Besides in environmental species, TLPQC-1 or variants thereof can be present in predominant pathogens of various genera including *Klebsiella pneumoniae* (Lee et al., 2016).

A *ftsH* protease xenolog is among the genes encoded by TLPQC-1. The FtsH protease belongs to the AAA (ATPase Associated with diverse cellular Activity) protease family that is universally conserved among eubacteria, mitochondria and chloroplasts (Ito and Akiyama, 2005). FtsH is anchored to the inner cell membrane at the N-terminus by two transmembrane helices, which flank a periplasmic domain; while the C-terminal cytosolic part consists of an AAA ATPase disaggregase and a M41-like endoprotease domain. The N-terminal domain is required for subunit interaction that affects the catalytic activities and enables processing of substrates from different compartments (Akiyama et al., 1998). Furthermore, the AAA ATPase domain binds, unfolds and translocates substrates into the proteolytic chamber. The enzymatic activity of the protease requires a Zn^2+^ ion coordinated by two conserved histidine residues in the ^417^HEXXH^421^ (X: any amino acid) motif and glutamate E^479^ to serve as a catalytic base (Tomoyasu et al., 1995; Ito and Akiyama, 2005; Bieniossek et al., 2006). The hexameric FtsH holoenzyme interacts with the modulator membrane proteins HflC and HflK to alter the proteolytic activity of FtsH (Kihara et al., 1996; Saikawa et al., 2004).

FtsH functionality has been extensively characterized in *Escherichia coli* K-12 mainly with respect to protein degradation (Schumann, 1999; Bittner et al., 2017). In this strain, FtsH controls protein quality by degrading out-of-context (membrane) proteins such as the subunit alpha of the F_1_F_0_ ATP synthase complex and the type 2 secretion system translocon protein SecY and contributes to the decision between lysis and lysogeny upon bacteriophage λ infection (Ito and Akiyama, 2005). Structurally, FtsH recognizes unfolded regions in proteins and aids in cleaning the cytoplasm from abnormal polypeptides tagged with the degradation signal SsrA added to truncated mRNAs (Herman et al., 1998). A major substrate of FtsH in *E. coli* K-12 is LpxC involved in biosynthesis of the lipid A anchor of LPS to maintain LPS homeostasis for optimal growth (Ogura et al., 1999). In addition, FtsH rapidly degrades the heat-shock transcription factor σ^32^ (RpoH) at non-stress temperatures (Herman et al., 1995; Tomoyasu et al., 1995).

Besides its function as a protease of unstructured and misfolded proteins, FtsH together with HflC, HflK, and YIdC acts as a chaperone to maintain the integrity of inner membrane proteins (Van Bloois et al., 2008). In yet another functionality, FtsH aids the translocation of the cytotoxic C-terminal domain of the tRNAase toxin colicin D from the periplasm into the cytoplasm (Walker et al., 2007).

FtsH functionality has hardly been explored in *P. aeruginosa* (Hinz et al., 2011; Langklotz et al., 2011). Here we show that *P. aeruginosa* clone C strain SG17M, besides the core genome homolog *ftsH1*, unconventionally encodes a *ftsH* xenolog on the clone C specific island PACGI-1. The core genome homolog *ftsH1* is required for optimal growth and contributes to a plethora of phenotypes such as antibiotic resistance, motility, biofilm formation, autolysis and production of secondary metabolites. The genomic island xenolog *ftsH2* mainly contributes to the same phenotypes in the *ftsH1* deletion background. Hetero-oligomer formation between FtsH1 and FtsH2 and the production of FtsH2 in the late stationary phase might provide novel functionalities and a unique role for FtsH2. The degradation of the heat shock sigma factor RpoH and processing of the phenazine biosynthesis protein PhzC are mainly FtsH1-dependent in *P. aeruginosa* SG17M.

## Results

### *P. aeruginosa* SG17M Is a Virulent Strain With an Unusual Regulation of the Type III Secretion System

The aquatic isolate SG17M is our reference clone C strain as it is the common assumption that environmental isolates infect patients (Römling et al., 1994; Martin et al., 2013). Indeed, SG17M was virulent in the non-mammalian model host *Galleria mellonella* larvae, although slightly less compared to the highly virulent reference strain *P. aeruginosa* PA14 (Miyata et al., 2003; Figure S1A). Surprisingly though, SG17M did not secrete effector proteins ExoS, ExoT, and ExoY of the type III secretion system (T3SS) under experimental conditions previously demonstrated to trigger type III secretion in *P. aeruginosa* (Toska et al., 2014; Figure S1B), despite that the invasive type SG17M codes for the T3SS locus and harbors the respective effector proteins. A panel of epidemiologically unrelated *P. aeruginosa* clone C and non-clone C isolates was subsequently tested for effector protein secretion (Figure S1C). While the reference *P. aeruginosa* strains, invasive PAO1 and cytotoxic PA14, secreted the respective effector proteins (Finck-Barbançon et al., 1997), lack of secretion was common among *P. aeruginosa* strains as several clinical and environmental clone C and non-clone C strains did not secrete T3SS effector proteins (Figure S1C). As expected, the outlier strain DSM1128, which does not encode a T3SS, did not secrete any effector proteins (Reboud et al., 2017; Figure S1C).

Biofilm formation is another common hallmark and pronounced virulence factor of *P. aeruginosa*. In steady state culture, SG17M displayed a voluminous unstructured biofilm with a relatively high percentage of dead cells, a characteristic also of other environmental isolates (Figure S2A). Although widely scattered, major biofilm parameters of SG17M such as roughness cluster with most other environmental and clinical clone C strains (Figure S2B; Römling et al., 2005), but were clearly distinct from most clone C cystic fibrosis isolates.

### A Xenolog of the FtsH Protease Is Present in Clone C Strain *P. aeruginosa* SG17M

**Figure 1:**
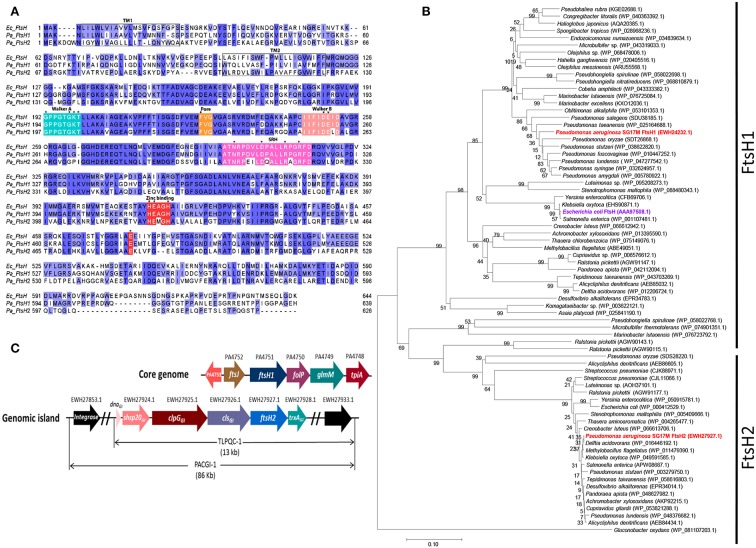
Homology, phylogeny and genomic context of FtsH1 and FtsH2. **(A)** Alignment of FtsH proteases from *E. coli* K12 and *P. aeruginosa* SG17M*. E. coli* FtsH (Ec_FtsH; AAA97508.1), *P. aeruginosa* SG17M core genome copy FtsH1 (Pa_FtsH1; EWH24232.1) and genomic island copy FtsH2 (Pa_FtsH2; EWH27927.1). Residues on a dark blue background indicate 100% conservation, while residues on a light blue background indicate 66% conservation. Different background colors indicate regions of functional motifs. Functional amino acids for ATP binding, substrate translocation and catalytic activity are labeled with an asterisk (^*^). Transmembrane segments TM1 and TM2 and the second region of homology (SRH) are also indicated. FtsH1 and FtsH2 of *P. aeruginosa* SG17M are 76 and 43% identical to *E. coli* FtsH, respectively. Sequences were aligned with MUSCLE using default settings (Edgar, 2004) and displayed in JalView (Waterhouse et al., 2009). **(B)** Phylogenetic analysis of closest homologs of FtsH1 and FtsH2 proteins from different taxonomic groups of bacteria. Examples of strains with multiple FtsH1/FtsH2 homologs are included in the tree: *Ralstonia pickettii* DTP0602 (4 homologs), *Alicycliphilus denitrificans* K601 (3 homologs), *Pseudomonas ludensis* (2 homologs), and *Streptococcus pneumoniae* SMRU2535 (3 homologs). *Gluconobacter oxydans* FtsH is included as outgroup. Branch lengths correspond to substitutions per site, bootstrap values are indicated in %. The sequence accession numbers are shown in parenthesis. **(C)** Neighboring genes flanking *ftsH1* on the core genome and *ftsH2* on the genomic island. Genes upstream of *ftsH1* are *ftsJ* (EWH24233.1) and a divergently transcribed *PA4752* encoding a conserved hypothetical protein (EWH24234.1), while genes downstream are *folP* (dihydropteroate synthase*-*EWH24231.1), *glmM* (phosphoglucosamine mutase*-*EWH24230.1), and *tpiA* (triosephosphate isomerase- EWH24229.1). Genes upstream of *ftsH2* are *cls* (cardiolipin synthase), disaggregase encoding *clp*G_*GI*_, small heat shock protein encoding *shsp20*_*GI*_ and transcription factor encoding *dn*a_*GI*_, whereas the downstream gene is *trx*A_*GI*_ (thioredoxin).

All *P. aeruginosa* strains encode, on the core genome, a homolog of the universal and functionally diverse metallo-protease FtsH (termed here FtsH1). The clone C specific genomic island PACGI-1 encodes the xenolog *ftsH2*. FtsH1 and FtsH2 share the characteristics of FtsH proteases with an N-terminal periplasmic domain flanked by transmembrane helices and followed by a highly conserved cytoplasmic AAA ATPase domain with an intact ATP binding Walker A/Walker B and a Second Region of Homology (SRH) motif. Furthermore, the pore motif MFVG required for coupling substrate unfolding and translocation (Krzywda et al., 2002) is equally present in the AAA ATPase domain. The M41 protease domain is characterized by the conserved Zn^2+^ binding motif “HEXXH” and harbors the catalytic base glutamic acid homologous to E_479_ of FtsH from *E. coli* (Tomoyasu et al., 1993b; Figure 1A). Core genome FtsH1 shows 76% sequence identity with the well-studied FtsH of *E. coli* K-12, while horizontally acquired FtsH2 is more distantly related to *P. aeruginosa* and *E. coli* K-12 core genome homologs with 45 and 43% sequence identity, respectively (Figures 1A,B and Figure S3). Indeed, FtsH2 proteins from unrelated species form a clearly distinct clade of highly similar proteins not congruent with the phylogenetic relationship of the species, while FtsH1 proteins cluster in congruence with species 16S RNA phylogeny (Figure 1B and Figure S3). The genes up- and down-stream of *ftsH1* and *ftsH2* are depicted in Figure 1C.

### Deletion of *ftsH* Genes Causes Growth Retardation

To investigate functionality of the core genome and the genomic island copy of *ftsH* in *P. aeruginosa* SG17M, we constructed deletion mutants of *ftsH1* and *ftsH2* and a double deletion mutant *ftsH1 ftsH2*. Comparison of the growth rate of wild type SG17M with the mutants in LB and M63 minimal medium at 37°C showed that the *ftsH1* mutant displayed a severe growth retardation both in LB and M63 medium (Figure S4). In LB medium, the doubling time of the *ftsH1* mutant was 0.9 h longer than the doubling time of the wild type which was 2.5 h. In M63 medium with citrate as a carbon source, the *ftsH1* mutant displayed a 1.5 h extended lag phase compared to the wild type with 4.5 h. Retardation of growth at both phases of the biphasic growth curve with the doubling times extended by 1.9 and 1.2 h compared to SG17M wild type doubling times of 2.5 and 2.2 h (Figure S4). In addition to an elongated doubling time, we observed a low optical density of the cell suspension in stationary phase in LB and M63 medium most likely due to the limited oxygen pressure. These findings indicate that the *ftsH1* mutant may only be viable due to a secondary mutation as observed in *E. coli* K-12 where viability of the *ftsH* mutant is ensured by a secondary mutation in *fabZ* coding for a key enzyme in fatty acid biosynthesis (Tomoyasu et al., 1993b; Ogura et al., 1999).

*FtsH2* mainly contributed to growth in the *ftsH1* deletion background in M63 medium. The lag phase was extended from 6 to 8 h and retardation of growth at the second phase of the biphasic growth curve led to a 1.5 h longer doubling time in the *ftsH1 ftsH2* double mutant compared to the *ftsH1* mutant (Figure S4A).

In conclusion, a complex contribution of *ftsH1* and, mainly in the absence of *ftsH1, ftsH2* to growth, was observed in SG17M. Associated with growth retardation, in *Borrelia burdorferi*, absence of *ftsH* causes clearly visible distortions of the membrane (Chu et al., 2016), while in *E. coli* K-12, deletion of *ftsH* leads to production of abnormal internal membranes at elevated temperatures (Ogura et al., 1999). No difference in cell morphology between wild type SG17M and *ftsH* mutants grown in LB medium was observed (Figure S5).

Growth retardation of the *ftsH1* mutant could be partially complemented with *ftsH1* expressed from the L-arabinose inducible plasmid pJN105 in LB and M63 medium (Figure S4B). Growth retardation of the *ftsH1 ftsH2* double mutant, in LB and, to a minor extent, in M63 medium, was partially complemented with *ftsH1* expressed from plasmid pJN105 (Figure S4D). Partial complementation with plasmid-encoded *ftsH2*, exceeding to some extent the degree of the contribution of the genomic island copy *ftsH2*, was observed in M63 medium (Figure S4D).

Of note, introduction of only the vector pJN105 caused growth retardation of the *ftsH2* mutant compared to plasmid-bearing wild type SG17M. Furthermore, this growth retardation could not be complemented by plasmid-borne *ftsH2*, but led to a more severe growth retardation phenotype, suggesting a substantial pJN105 vector effect in the *ftsH2* mutant background.

In an alternative experimental set-up on LB agar at 37°C, growth retardation of the *ftsH1* mutant was also complemented with pJN105 encoded *ftsH1* (Figure S6). While growth retardation was not observed for the *ftsH2* mutant (Figure S6), growth of the *ftsH1 ftsH2* double mutant was decreased compared to the *ftsH1* mutant. In line with the functionality observed in the single deletion mutants, the *ftsH1 ftsH2* double mutant was complemented with *ftsH1* and, partially, with *ftsH2* expressed from pJN105 indicating again that *ftsH2* can partly compensate for *ftsH1* functionality in the *ftsH1* mutant background (Figure S6).

### FtsH1 and FtsH2 Have Distinct Production Patterns Throughout the Growth Curve

**Figure 2:**
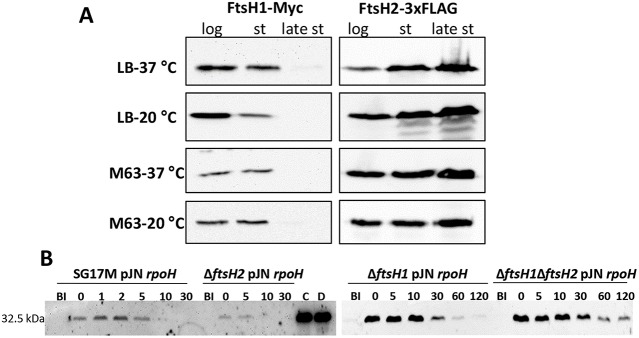
Differential production of FtsH1 and FtsH2 during the growth phase and *in vivo* degradation of the sigma factor RpoH by FtsH proteases in *P. aeruginosa* SG17M. **(A)** Production of FtsH1-Myc and FtsH2-3xFLAG assessed in the logarithmic (log), stationary (st) and late stationary phase (late st) at 37 and 20°C in LB and M63 medium. In LB medium, the logarithmic phase equals OD_600_ = 1 and late stationary phase OD_600_ = 4 (26 h at 37°C; 47 h at 20°C). Stationary phase in LB medium equals OD_600_ = 2.5 at 20°C (18 h) and OD_600_ = 4.5 at 37°C (8 h). In M63 medium at both temperatures, logarithmic phase equals OD_600_ = 0.25, stationary phase OD_600_ = 0.6 (8 h at 37°C; 30 h at 20°C) late stationary phase OD_600_ = 0.8 (26 h at 37°C; 120 h at 20°C). One blot of two independent biological replicates with congruent results is shown. **(B)** 6xHis-RpoH degradation after plasmid-borne induction for 30 min as assessed by Western blotting in the wild type *P. aeruginosa* SG17M, Δ*ftsH2*, Δ*ftsH1*, and Δ*ftsH1*Δ*ftsH2* background. BI (Before Induction) refers to the sample before a 30 min expression of *rpoH* from plasmid pJN105 with 1% L-arabinose. Numbers above the blot represent time in min after termination of translation with 300 μg ml^−1^ spectinomycin. Lanes marked with “C” and “D” represent the 6xHis-RpoH signal at time point = 0 min in the Δ*ftsH1* and Δ*ftsH1*Δ*ftsH2* background, respectively. One blot of two independent biological replicates with congruent results is shown.

The *ftsH2* mutant did not show a pronounced growth retardation phenotype (Figure S4A). As genes on horizontally transferred genomic islands are frequently silenced (Perez-Rueda and Ibarra, 2015), we were wondering whether FtsH2 is produced in comparison to FtsH1. Investigation of protein expression in LB and M63 minimal medium at 20 and 37°C showed that chromosomally encoded FtsH1-Myc was produced in the logarithmic and the stationary phase, but was absent in the late stationary phase (Figure 2A). In contrast, chromosomally encoded FtsH2-3xFLAG was constitutively produced in all growth phases with a rise in production in late stationary phase (Figure 2A) suggesting an exclusive biological function of FtsH2 in the late stationary phase. These findings are in agreement with negligible transcriptional activity for *ftsH1* in the stationary phase of growth as assessed in *P. aeruginosa* PAO (Tavares et al., 2003).

### The Heat Shock Transcription Factor RpoH Is Degraded by FtsH Proteases in *P. aeruginosa*

**Figure 3:**
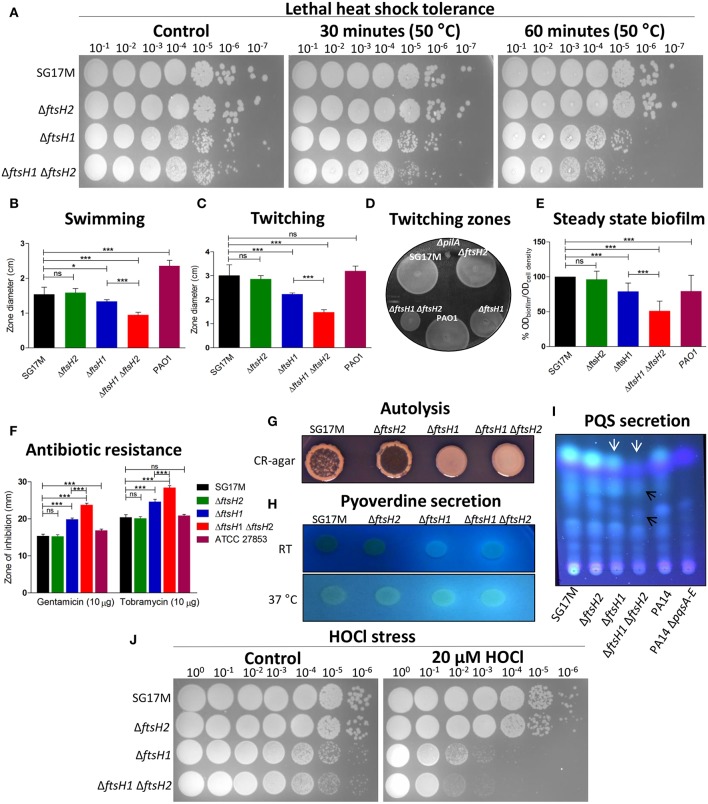
Bacterial phenotypes affected by *ftsH1* and/or *ftsH2* in *P. aeruginosa* SG17M. The proteases contribute to tolerance toward lethal heat stress, flagella and type IV pili mediated motility, biofilm formation, antibiotic resistance, autolysis, pyoverdine secretion, PQS secretion, and resistance against hypochlorous acid (HOCl). **(A)** Lethal heat shock was conducted at 50°C for 30 and 60 min, serial dilutions were spotted on LB agar and incubated for 24 h. **(B)** Flagella-dependent swimming motility was tested at 37°C. The mean value was calculated from six independent experiments and error bars indicate SD (^*^P < 0.05 and ^***^P < 0.0001). **(C)** Pili-dependent twitching motility was tested at 37°C. The mean value was calculated from eight independent experiments and error bars indicate SD (^***^P < 0.0001). **(D)** Zones of twitching motility on the bottom of a plastic petri plate stained with TEM developer solution (glacial acetic acid: methanol: water, 1:5:4 ratio). *Pseudomonas aeruginosa* PAO1 Δ*pilA* was the negative control. **(E)** Relative biofilm formation in 96-well plate was assessed in LB medium at 37°C. *P. aeruginosa* PAO1 is a positive control. The mean value was calculated from two independent experiments in 32 technical replicates and error bars indicate SD (^***^P < 0.0001). **(F)** Resistance against aminoglycosides (gentamicin and tobramycin) was tested using the disk diffusion test. *P. aeruginosa* ATCC 27853 was the reference strain. The mean value was calculated from two independent experiments with 12 technical replicates and error bars indicate SD (^***^P < 0.0001)). **(G)** Autolysis was assessed on Congo Red (CR) medium at room temperature (21°C) after 5 days of incubation. **(H)** Pyoverdine production was tested on LB agar at room temperature (21°C) and 37°C. **(I)** PQS molecules were extracted from equal cell number at stationary phase growing at 37°C and run on thin layer chromatography (TLC) plate, PQS bands (white arrows) and affected PQS precursors (black arrows) were visualized with UV 312 nm. *P. aeruginosa* PA14 and PA14 Δ*pqsA-E* were used as a positive and negative control, respectively. **(J)** Oxidative stress by HOCl was conducted by incubating the cells with 20 μM HOCl for 30 min at 37°C. Serial dilutions were spotted on LB agar and incubated for 20 h.

Rapid RpoH degradation under non-heat-shock conditions is FtsH-dependent in *E. coli* (Herman et al., 1995; Bittner et al., 2017), however degradation of RpoH by FtsH has never been tested in *P. aeruginosa*. After 30 min of induction, the steady state levels of RpoH produced from pJN105 were significantly higher in the *ftsH1* mutant and *ftsH1 ftsH2* double mutant background than in the wild type SG17M. RpoH was degraded rapidly in the wild type and *ftsH2* deletion mutant background with a half-life of <10 min (Figure 2B). RpoH was, however, more slowly degraded with an estimated half-life between 10 and 30 min in the *ftsH1* mutant and *ftsH1 ftsH2* double mutant background indicating that *ftsH1* encodes the protease that mainly degrades RpoH (Figure 2B). In agreement with a deregulated heat shock response, deletion of *ftsH1*, but not *ftsH2* moderately enhanced sensitivity to a lethal heat shock of 50°C for 30 and 60 min (Figure 3A). The double deletion mutant *ftsH1 ftsH2*, though, showed an equal lethal heat shock sensitivity, but a smaller colony size upon 60 min of lethal heat shock.

### FtsH1 and FtsH2 Contribute to Motility

As ftsH is a multifunctional protease, we wondered whether *ftsH1* and *ftsH2* contribute to alternative phenotypes besides growth and heat shock. *FtsH* had no effect on the unconventional T3SS effector secretion, though (data not shown). Assessment of flagella-dependent swimming motility of SG17M and *ftsH* mutants at 37°C showed that deletion of *ftsH2* did not affect swimming motility. However, deletion of *ftsH1* caused a 13% reduction in swimming motility, which was, with 38% reduction, more pronounced in the *ftsH1 ftsH2* double deletion background (Figure 3B). Similarly, type IV pili-dependent twitching motility at 37°C was not affected by deletion of *ftsH2*. However, deletion of *ftsH1*, though, reduced twitching motility by 26%, which was, with 51% reduction, again more pronounced in the *ftsH1 ftsH2* double deletion background (Figures 3C,D). The swimming and twitching motility defects in the *ftsH1* mutant and *ftsH1 ftsH2* double mutant were complemented by overexpression of *ftsH1* and, partially, by overexpression of *ftsH2* from the pJN105 vector (Figures S7A,B).

### FtsH1 and FtsH2 Contribute to Biofilm Development

Biofilm formation is a major virulence phenotype of *P. aeruginosa*. The cell density normalized steady-state culture biofilm of SG17M developed in a polystyrene microtiter tray after 24 h was not affected upon deletion of *ftsH2*. However, deletion of *ftsH1* led to a 30% lower relative biofilm formation (Figure 3E), while the *ftsH1 ftsH2* double mutant formed 60% less cell density normalized biofilm compared to SG17M (Figure 3E).

**Figure 4:**
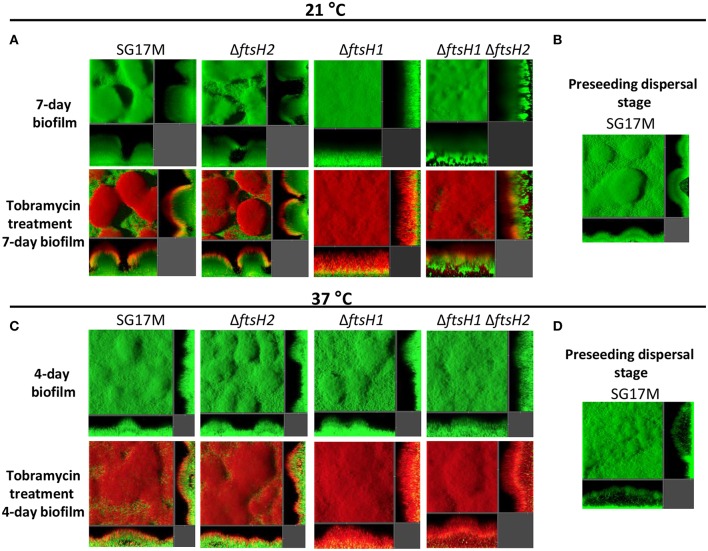
Contribution of *P. aeruginosa* SG17M *ftsH* gene products to biofilm architecture and tobramycin tolerance in a microfluidic flow cell chamber biofilm. **(A)** Biofilm formation of *P. aeruginosa* SG17M and *ftsH* mutants at 21°C after 7 days (top panel) and application of tobramycin for 20 h on the mature 7-day old biofilm (bottom panel). **(B)** Preseeding dispersal stage of SG17M at 21°C. **(C)** Biofilm formation of SG17M and *ftsH* mutants at 37°C after 4 day (top panel) and application of tobramycin for 20 h on the mature 4-day old biofilm (bottom panel). **(D)** Preseeding dispersal stage of SG17M at 37°C. Strains are tagged with GFP and propidium iodide (PI) was used to stain dead bacteria.

Biofilm formation was also assessed in a micro fluidics flow chamber at 21 and 37°C. At 21°C, the wild type SG17M formed a characteristic *P. aeruginosa* biofilm with mushroom-like microcolonies (Figure 4A). While *ftsH2* had no effect on the amount and structure of the biofilm, the *ftsH1* mutant formed an undifferentiated flat biofilm (Figure 4A). Of note, the *ftsH1 ftsH2* double mutant formed previously uncharacterized dense irregular biofilm structures anchored to the substratum at a few contact points (Figure 4A) indicating that *ftsH2* contributes to flow cell biofilm formation in the absence of *ftsH1*. Furthermore, the biofilm of wild type SG17M (representative picture in Figure 4B) and the *ftsH2* mutant formed preseeding dispersal structures filled with motile bacteria (Videos S1, S2). These structures were absent in the biofilm formed by the *ftsH1* mutant and *ftsH1 ftsH2* double mutant (data not shown).

At 37°C, the biofilm structures formed by SG17M and *ftsH* mutant derivatives after 4 days were different. Wild type SG17M and all *ftsH* mutants formed less pronounced mushroom structures (Figure 4C), but large structures with internal voids filled with motile bacteria (preseeding dispersal) were present. These voids were more frequent and bigger than those observed at 21°C [Figure 4C with representative picture of the wild type SG17M (Figure 4D)].

### FtsH1 and FtsH2 Contribute to Aminoglycoside Tolerance in Planktonic Culture and in Biofilms

Previously, it has been shown that the single copy of *ftsH* contributes to antibiotic resistance in the reference strain *P. aeruginosa* PAO1 (Hinz et al., 2011). Among different classes of antibiotics tested, enhanced sensitivity was only observed against the aminoglycosides gentamicin and tobramycin (Gibson et al., 2003), clinically relevant to treat *P. aeruginosa* infections. The *ftsH1* mutant showed a 29.4 and 20.4% extended zone of inhibition with gentamicin and tobramycin, respectively, as compared to the wild type SG17M (Figure 3F). The contribution of *ftsH2* to the phenotype led to a 54.9 and 39.2% extended zone of inhibition in the *ftsH1 ftsH2* double mutant as compared to the wild type SG17M (Figure 3F). The extended zone of inhibition of the *ftsH1 ftsH2* double mutant against aminoglycoside antibiotics was reduced upon overexpression of *ftsH1*, and to a minor extent upon overexpression of *ftsH2* from plasmid pJN105 (Figure S7C).

Subsequently, we tested the tolerance profile of biofilms in a micro fluidics flow chamber against tobramycin. In the wild type SG17M, the *ftsH2* mutant and, despite possessing a different biofilm structure, the *ftsH1* mutant, dead cells were only observed at the top of the biofilm after long-term tobramycin treatment for 20 h at 21°C (Figure 4A). The ratio of dead to life cells was significantly higher in the *ftsH1* mutant as compared to wild type SG17M. The *ftsH1 ftsH2* double mutant showed a reduced zone of dead cells compared to the *ftsH1* mutant at the top of the biofilm, but also cells at the biofilm-glass interface were not viable (Figure 4A).

Although wild type and *ftsH* mutants displayed similar biofilm structures at 37°C, the tobramycin tolerance profile showed differences. The *ftsH1* mutant displayed reduced tolerance as indicated by the higher ratio of dead to live cells compared to the wild type SG17M (Figure 4C). The *ftsH1 ftsH2* double mutant showed a further reduced tolerance than the *ftsH1* mutant indicating again a role of *ftsH2* in antibiotic tolerance in the absence of *ftsH1* (Figure 4C).

### FtsH1 Regulates Autolysis

Cellular autolysis optimizes fitness of the bacterial community structure by disposing less fit members that cannot tolerate stress (D′Argenio et al., 2002; Häussler and Becker, 2008). We found autolysis to be a distinct phenotype of agar-grown colonies of the wild type SG17M grown at low temperature of 21°C for 5 days. *FtsH1* promoted autolysis as the *ftsH2* mutant, but not the *ftsH1* mutant and the *ftsH1 ftsH2* double mutant, showed strong autolysis similar to SG17M (Figure 3G).

Other common strains of *P. aeruginosa* such as the reference strain PA14, a clinical isolate, show a biofilm phenotype upon plate growth at low temperature (Friedman and Kolter, 2004). Colony autolysis has been observed previously in a *P. aeruginosa* quinolone signal (PQS) overproduction mutant (D′Argenio et al., 2002). To investigate the abundance of the autolysis phenotype, we tested a selected panel of clone C and non-clone C strains of environmental and clinical origin for colony morphology and autolysis at 21, 28, and 37°C (Figure S8A). Although a diversity of colony morphotypes including biofilm morphotypes were observed, consistently, autolysis was almost exclusively a trait of clone C strains of environmental origin at 21°C (Figure S8A).

### FtsH1 and FtsH2 Regulate Pyoverdine and PQS Production

Pyoverdines (PVDs) are a group of green fluorescent siderophores that have a dual-role in iron uptake and pathogenesis (Visca et al., 2007; Schalk and Guillon, 2013). Pyoverdine secretion was evaluated on LB agar at 21 and 37°C. In contrast to well-established strains of *P. aeruginosa* PAO1 and PA14 (Figure S8A), pyoverdine was not secreted by the wild type SG17M and its *ftsH2* mutant (Figure 3H). However, pyoverdine secretion was stimulated upon *ftsH1* deletion, which was more pronounced in the *ftsH1 ftsH2* double deletion mutant. Overexpression of *ftsH1* from plasmid pJN105 at 37°C turned off secretion of pyoverdine in the *ftsH1* and *ftsH1 ftsH2* deletion mutant (Figure S8B).

Furthermore, we were wondering whether this unconventional pattern of pyoverdine secretion is common among *P. aeruginosa* isolates. The selected panel of clone C and non-clone C strains of environmental and clinical origin showed that environmental isolates of clone C rarely secreted pyoverdine under the applied experimental conditions (Figure S8A).

PQS is a secondary metabolite and quorum sensing molecule which is produced by *P. aeruginosa* including strain SG17M (Figure 3I). Deletion of *ftsH2* had no effect on PQS secretion whereas in the *ftsH1* mutant PQS was attenuated. The double deletion mutant *ftsH1 ftsH2* did not produce PQS but perhaps minute amounts of precursor molecules (remaining signals were not further characterized). Near absence of PQS production as observed in the *ftsH1 ftsH2* double mutant has previously been associated with alterations in the amount of biofilm, its structure, and enhanced susceptibility against the aminoglycoside antibiotic tobramycin (Yang et al., 2009; Chiang et al., 2013).

### FtsH1 and FtsH2 Contribute to HOCl Oxidative Stress Tolerance

Hypohalites are effective reactive defense molecules of neutrophils, major innate immune cells against bacterial infections. Deletion of *ftsH1* not only sensitized against 20 μM HOCl, but also led to a smaller colony size upon regrowth, while the double deletion mutant *ftsH1 ftsH2* displayed a higher sensitivity toward this hypohalite. The single deletion mutant *ftsH2* did not show increased sensitivity toward oxidative stress as compared to the wild type SG17M (Figure 3J).

### FtsH1 and FtsH2 Form Homo- and Hetero-Oligomers

**Figure 5:**
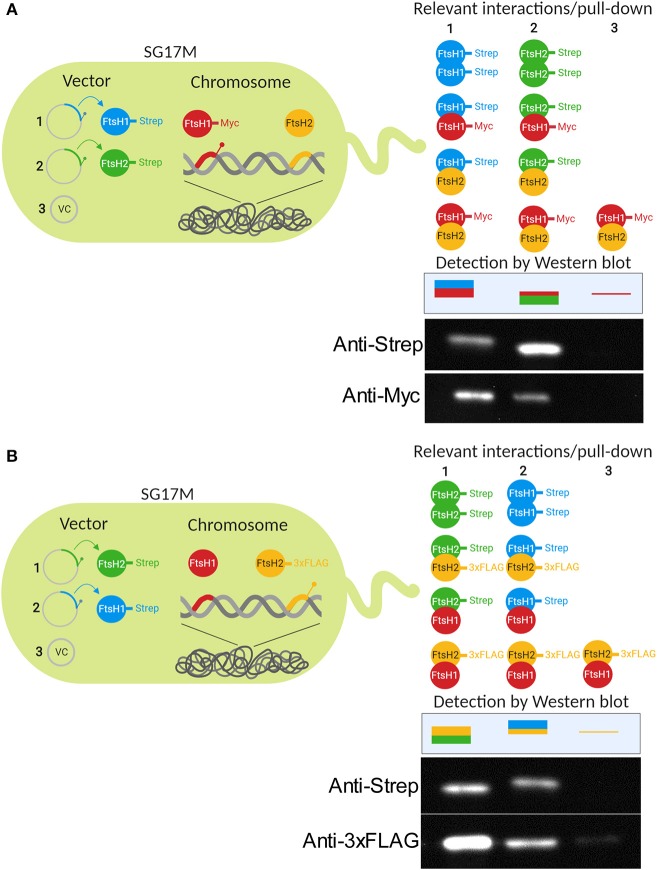
FtsH1 and FtsH2 can form homo- and hetero-oligomers in *P. aeruginosa* SG17M. Homo- and hetero-oligomer formation of FtsH proteases was tested in pull-down assay using cells in the logarithmic growth phase at 37°C in SG17M in the FtsH1-Myc background **(A)** and FtsH2-3xFLAG background **(B)**. Pull-down was performed with FtsH1-Strep or FtsH2-Strep produced from plasmid pJN105. Negative control for the pull down assay was the empty pJN105 vector. Detection of proteins was performed by Western blot analysis using antibodies against the Strep-, Myc-, and 3xFLAG-tag. The sketch demonstrates the experimental set-up (left) and the relevant potential interactions between FtsH proteins and observed Western blot signals for the two tags (right). Figure created with BioRender.

The FtsH protease arranges in hexamers with its cytosolic parts to form a barrel-like structure in which proteolysis takes place (Tomoyasu et al., 1993a). As genetic data suggested interactions between FtsH1 and FtsH2, *in vivo* interaction between FtsH1 and FtsH2 was tested. We created a strain where chromosomally encoded FtsH1-Myc was co-produced with either Strep-tagged FtsH1 or FtsH2 (Figure 5A). In these strains, plasmid-produced FtsH1-Strep and FtsH2-Strep pulled down FtsH1-Myc suggesting hetero-oligomer formation *in vivo*. Specificity of the pull-down assay was demonstrated as no signal was detected for FtsH1-Myc with an empty vector control. In a strain with chromosomally encoded FtsH2-3xFLAG (Figure 5B), FtsH2-3xFLAG co-precipitated not only with plasmid-expressed FtsH2-Strep, but also with FtsH1-Strep. A faint background signal could be detected for FtsH2-3xFLAG with the empty vector control. To further support evidence for hetero-oligomer formation, FtsH1 and FtsH2 were pulled down reciprocally when probing for substrate interaction (Table S2, see below). Thus as a conclusion, experimental evidence suggests that homo- and hetero-oligomers can be formed *in vivo* which might create protein complexes with novel substrate specificity and/or regulatory features.

### PhzC Processing Is Dependent on FtsH Proteases in *P. aeruginosa* SG17M

FtsH1 and FtsH2 showed different substrate specificities with respect to RpoH degradation (Figure 2B). To identify additional substrates for FtsH1 and FtsH2, a trapping approach was applied with FtsH variants FtsH1^trap^ and FtsH2^trap^ tagged with a C-terminal Strep-tag. These variants are proficient in ATPase activity and substrate binding, but with a catalytically inactive protease due to a H416Y and H420Y amino acid exchange in the zinc binding HEXXH-motif of FtsH1 and FtsH2, respectively. Subsequently, interacting proteins were cross-linked with plasmid-expressed FtsH^trap^, complexes were pulled down and proteins were separated on SDS-PAGE gels without and after reversal of the cross-link. Candidate interacting protein bands from biological and technical replicates, reproducibly detected on the SDS-PAGE gel (and not present in the vector control; Figure S9) were cut, digested with trypsin and subsequently identified by mass spectrometry (Flynn et al., 2003; Westphal et al., 2012). As a detection control, bands representing FtsH1^trap^ and FtsH2^*trap*^ were also cut out (FtsH1^trap^ was discovered with protein coverage/peptides detected: 16%/9 and for FtsH2^trap^, the coverage was 40% with 24 peptides detected). Proteins detected with the most abundant peptide fragments and highest coverage are listed in Table S2.

Of note, the FtsH modulator protein HflC was found as a candidate interaction partner for FtsH1 and FtsH2. This is consistent with the FtsH hexamer to interact with membrane proteins HflC and HflK in *E. coli* K-12 (Kihara et al., 1996). Furthermore, the multifactorial membrane protein ATP synthase subunit alpha, a substrate for FtsH in *E. coli* (Akiyama et al., 1996), was also pulled-down with FtsH1 and FtsH2. Although this analysis was limited, pull down of distinct proteins associated with FtsH1 and FtsH2 suggests distinct functionality for the two proteins (Table S2).

**Figure 6:**
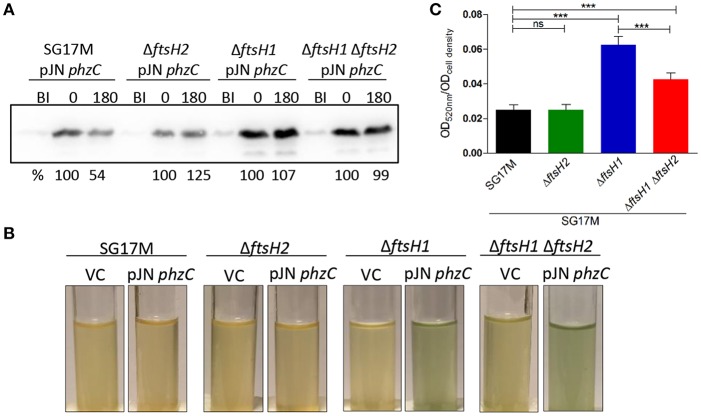
Degradation of PhzC-6xHis is *ftsH1*-dependent in *P. aeruginosa* SG17M. **(A)** *In vivo* degradation assay at 37°C in the logarithmic growth phase. BI (Before Induction) refers to the sample before inducing the production of PhzC. Numbers above the blot represent time in min after the termination of translation with 300 μg ml^−1^ spectinomycin following 30 min induction with 0.1% L-arabinose. The signal intensity of PhzC-6xHis at time 0 is set 100%. Band intensities after 10 s exposure were quantified using the software ImageJ. One representative experiment from two independent biological replicates with congruent results is shown. **(B)** Accumulation of pyocyanin pigment documented after expression of *phzC* for 30 min at 37°C followed by 180 min incubation after termination of translation with 300 μg ml^−1^ spectinomycin. Strains harboring vector control (VC) were used for comparison. **(C)** Pyocyanin extraction and quantification normalized to cell density from cultures grown for 20 h at 37°C. The mean value was calculated from two independent experiments with six technical replicates. Error bars indicate SD (^***^P < 0.0001).

Selectively found to be pulled down with FtsH1 were PhzC and PhzF, two proteins encoded by the phenazine biosynthesis operon, suggesting a role of FtsH1 in regulation of phenazine biosynthesis. *P. aeruginosa* harbors two phenazine biosynthesis operons encoding identical PhzC proteins. To confirm PhzC to be a substrate of FtsH1, we assessed degradation of PhzC similar as for RpoH (Figures 2B, 6A). After induction of protein production with 0.1% L-arabinose for 30 min and subsequent spectinomycin treatment to stop translation, ~50% of PhzC was degraded after 3 h in the wild type background, whereas PhzC was stable in all mutant backgrounds (Figure 6A and Figure S10). In support of this observation, production of PhzC in the *ftsH1* mutant and *ftsH1 ftsH2* double deletion mutant compared to the wild type was associated with the development of an intense green color in the medium due to pyocyanin accumulation (Figure 6B).

Extraction of pyocyanin from overnight cultures grown at 37°C showed that, while the wild type and the *ftsH2* mutant showed similar relative pyocyanin levels (Figure 6C), the *ftsH1* and the *ftsH1 ftsH2* double mutant accumulated three- and twofold more pyocyanin (Figure 6C) confirming again a contribution of *ftsH1* and, conditionally, *ftsH2* in the *ftsH1* background to processing of PhzC.

## Discussion

**Figure 7:**
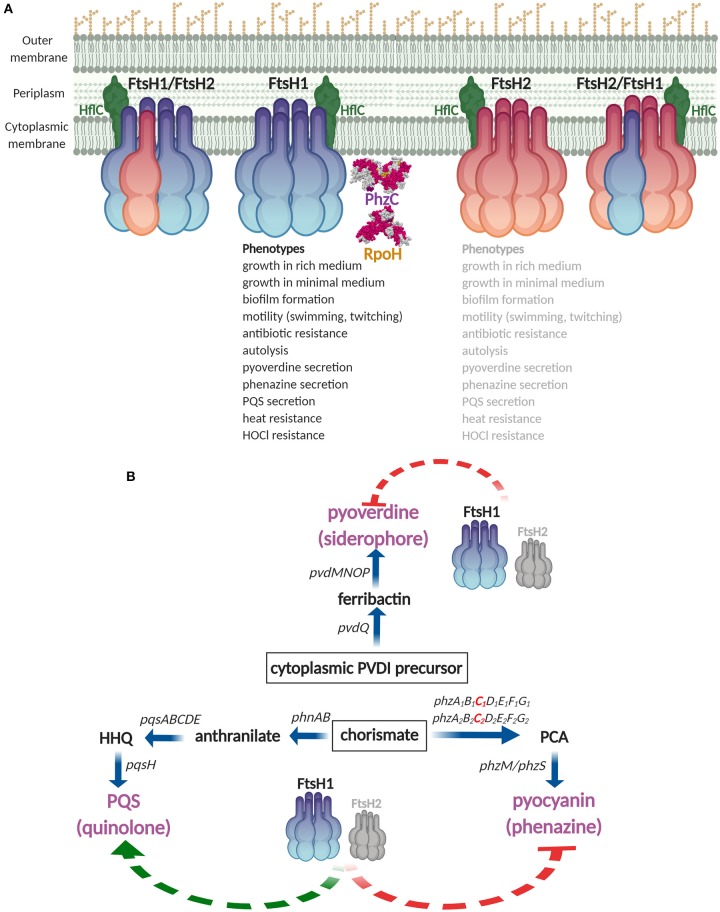
Summary diagram of FtsH1 and FtsH2 functionality in *P. aeruginosa* SG17M. **(A)** FtsH anchored to the inner cell membrane as homo- and hetero-oligomers are represented by different color codes for monomers. Phenotypes are affected by FtsH1 (listed in black), with back-up contribution of FtsH2 (listed in gray). FtsH1/FtsH2 associated membrane protein HflC and confirmed substrates, RpoH and PhzC, are indicated. RpoH and PhzC structure models are obtained from PDB entry 1SIG (Narberhaus and Balsiger, 2003) and 6BMC (Sterritt et al., 2018), respectively. **(B)** FtsH1 and FtsH2 act as major regulators of secondary metabolites by affecting the secretion of pyoverdine, pyocyanin, and *Pseudomonas* quinolone signal PQS. HHQ, PQS intermediate 2-heptyl-4-quinolone; PCA, phenazine intermediate phenazine-1-carboxylic acid. Figure created with BioRender.

In this work, we initially characterized the multifunctional FtsH proteases in *P. aeruginosa* clone C strain SG17M. Deletion of core genome *ftsH1* affected a variety of phenotypes, while the genomic island copy *ftsH2* showed a cumulative effect in the *ftsH1* mutant background (Figure 7A). The core genome protease homolog *ftsH1* promotes optimal growth (Figure S4) and, although we have not rigorously confirmed all phenotypes by complementation, *ftsH1* is required for expression of a multitude of unrelated phenotypes from motility to secondary metabolite production and to-be-characterized autolysis and HOCl resistance (Figures 3, 4 and Figures S6, S7, S8B). Besides the heat shock sigma factor RpoH, a known target of FtsH, PhzC, channeling precursor molecules for phenazine biosynthesis, was identified as a novel substrate for the FtsH1 protease. This cumulative functionality of FtsH1 and FtsH2 with broadly unequal manifestation, which could be based on the formation of hetero-oligomers, was evident by more efficient complementation of selected phenotypes with *ftsH1* than *ftsH2* in the double deletion mutant. Distinct expression patterns of FtsH proteins and the potential formation of homo- and hetero-oligomers indicate future discovery of novel substrate specificities and functionalities.

The *ftsH1* deletion mutant of *P. aeruginosa* SG17M showed a severe growth retardation phenotype. Essentiality and effect on growth are often characteristics of FtsH proteases in various bacterial species (Bittner et al., 2017). In the Lyme disease pathogen *Borrelia burgdorferi, ftsH* deletion causes cell death, membrane deformation and impaired infectivity (Chu et al., 2016). However, the molecular basis of growth promotion seems to be different in different organisms. The *ftsH* deletion mutant in *E. coli* is non-viable due to deregulation of LpxC catalyzing the key step in LPS biosynthesis, which leads to disturbance of the LPS/phospholipid ratio in the outer membrane and aberrant membrane production (Ogura et al., 1999). Alternative mechanisms triggering growth retardation must occur in *P. aeruginosa* SG17M as LpxC has been shown to be a poor substrate for FtsH in *P. aeruginosa*, consequently its accumulation is not contributing to toxicity (Langklotz et al., 2011). Furthermore, with LB as growth medium, membrane distortions are not observed (Figure S5). Thus, most likely, other protease(s) are regulating LPS homeostasis through LpxC in *P. aeruginosa*. In alpha-proteobacteria, for example, degradation of LpxC is evolutionary shifted from FtsH to the Lon protease (Langklotz et al., 2011).

FtsH1 is a major protease degrading RpoH in *P. aeruginosa* SG17M (Figures 2B, 7A), similarly as in *E. coli* (Herman et al., 1995). Although deregulation of the heat shock response through the heat shock sigma factor RpoH and a subsequent activation of e.g., proteases, could be deleterious in *P. aeruginosa* SG17M, we have no indications that overexpression of RpoH retards growth (data not shown). Thus the molecular basis of pronounced growth retardation in *P. aeruginosa* upon deletion of *ftsH1* remains to be unraveled.

Conventionally, a *ftsH* homolog is present on the *P. aeruginosa* core genome, while *P. aeruginosa* clone C strain SG17M encodes an additional xenolog on the PACGI-1/TLPQC-1 genomic island. Several organisms code for more than one FtsH protease with hetero-oligomerization among homologs being the rule rather than the exception. For example, the cyanobacterium *Synechocystis* sp. PCC 6803 has four FtsH homologs (FtsH1-FtsH4) (Sacharz et al., 2015). While FtsH1 and FtsH3 contribute to cell viability (Boehm et al., 2012), hetero-complexes serve distinct functions, such as being involved in iron homeostasis through affecting the availability of the iron transcription regulator Fur (Krynická et al., 2014). In eukaryotes, the *Arabidopsis* chloroplast codes for 12 *ftsH* homologs, which are, for example, involved in photosynthesis (Nishimura et al., 2016). Furthermore, humans possess three mitochondrial homologs of *ftsH* (Janska et al., 2013). A hallmark of multicopy *ftsH* genes is thus a differential functionality. With two homologs in clone C strains, the functionality of FtsH proteases is expected to be increased and/or widened through FtsH2 homo-oligomer and even FtsH1/FtsH2 hetero-oligomer formation.

FtsH2 encoded on TLPQC-1/PACGI-1 is constitutively expressed throughout different growth phases with elevated expression in the late stationary phase (Figure 2A). The exclusive production of FtsH2 in the late stationary phase of growth compared to the core genome copy FtsH1, which is not expressed in the late stationary phase (Figure 2A; Tavares et al., 2003), suggests a yet-to-be-discovered unique role for FtsH2 in the stationary phase, which could also aid the promotion of horizontal transfer of PACGI-1. Of note, also other gene products encoded on the TLPQC-1 locus, i.e., the small heat shock protein sHsp20_GI_ and the disaggregase ClpG_GI_ that confers lethal heat shock tolerance, are unconventionally and predominantly expressed in the stationary growth phase (Lee et al., 2015, 2018). While our studies have clearly demonstrated advantageous phenotypes for several PACGI-1 gene products in the environmental strain *P. aeruginosa* SG17M, an advantage of PACGI-1 in other strain backgrounds is less clear (Klockgether et al., 2013). We can, though, envisage a scenario where inhibition of FtsH1, e.g., through phage proteins (Kobiler et al., 2002) during antibiotic treatment promotes the acquisition and/or maintenance of PACGI-1/TLPQC-1.

Our trapping approach of substrate identification for FtsH proteins was validated by recovery of the modulator protein HflC that has been demonstrated to form a complex with FtsH in the cell membrane in *E. coli* (Kihara et al., 1996) (Figure 7A and Table S2). Additionally, the subunit alpha of the multisubunit F_1_ F_0_ ATP synthase complex is a FtsH substrate in *E. coli* (Akiyama et al., 1996) (Table S2). PhzC was verified as a novel protein interacting with the FtsH1 protease (Figures 6A, 7A and Table S2) with the phenazine end product pyocyanin to accumulate in the medium in the *ftsH1* and the *ftsH1 ftsH2* double mutant (Figure 6B). The degradation experiment indicated PhzC to be a substrate of also FtsH2, however, as pyocyanin accumulation was not enhanced in the *ftsH2* mutant, this result requires further confirmation. Phenazines are multipurpose redox-recycling signaling antibiotics involved in a variety of biological functions such as biofilm formation and cytotoxicity against eukaryotes (Pierson and Pierson, 2010; Cezairliyan et al., 2013; Das et al., 2015; Meirelles and Newman, 2018). *P. aeruginosa* harbors two phenazine biosynthesis operons with identical PhzC and PhzF sequences. Of note, this study identified the FtsH protease as a regulatory bottleneck to restrict biosynthesis of this potentially auto-poisoning substance produced by two differentially regulated biosynthesis operons (Recinos et al., 2012). In summary, regulation of secretion of the secondary metabolites pyocyanin, pyoverdine, and PQS indicates a novel role for the FtsH protease as a major regulator of secondary metabolites/low molecular weight virulence factors in this environmental isolate of *P. aeruginosa* clone C (Figure 7B).

In conclusion, the core genome homolog *ftsH1* promotes fitness and adaptation of *P. aeruginosa* SG17M and potentially interacts with the horizontally acquired genomic island homolog *ftsH2*, which provides additive advantages in the absence of *ftsH1* to clone C, one of the most predominant clones of *P. aeruginosa*.

## Experimental Procedures

### Strains and Growth Conditions

Strains used in this study are described in Table 1. Strains were routinely cultivated aerobically at 37°C in Luria-Bertani (LB) broth medium (BD Difco) unless otherwise indicated. Other media used were M63-citrate minimal medium (Hinsa and O′Toole, 2006), Congo Red (CR) agar (Römling et al., 1998) and Vogel-Bonner minimal medium (VBMM) (Choi and Schweizer, 2005). If needed, 30 μg ml^−1^ gentamicin (Gm) and 70 μg ml^−1^ tetracycline (Tc) were used for *P. aeruginosa* SG17M. *Escherichia coli* TOP10 was used to propagate recombinant plasmids with antibiotics Gm, Tc, and kanamycin (Km) at 30, 20, and 100 μg ml^−1^, respectively.

**Table 1:** Bacterial strains used in this study.

**Strain**	**Genotype/Source**	**References**
***P. aeruginosa*** **SG17M and derivatives**		
SG17M	Environment, river water	Römling et al., 1994
SG17M051	SG17M Δ*ftsH1*	This study
SG17M052	SG17M Δ*ftsH2*	This study
SG17M053	SG17M Δ*ftsH1*Δ*ftsH2*	This study
SG17M054	SG17M *ftsH1*-*Myc*	This study
SG17M055	SG17M *ftsH2-3xFLAG*	This study
SG17M056	SG17M051 *att*Tn*7*::P*araC-ftsH1*	This study
SG17M057	SG17M052 *att*Tn*7*::P*araC-ftsH2*	This study
SG17M058	SG17M053 *att*Tn*7*::P*araC-ftsH1*	This study
SG17M059	SG17M053 *att*Tn*7*::P*araC-ftsH2*	This study
SG17M060	SG17M Δ*exs*	Lee et al., 2015
SG17M-gfp	SG17M *att*Tn*7*::*gfp-gm*	This study
SG17M051-gfp	SG17M051 *att*Tn*7*::*gfp-gm*	This study
SG17M052-gfp	SG17M052 *att*Tn*7*::*gfp-gm*	This study
SG17M053-gfp	SG17M053 *att*Tn*7*::*gfp-gm*	This study
**Other** ***P. aeruginosa*** **strains**		
PAO1 (DSM1707)	Wild type	Holloway, 1955
PAO1-051	PAO1 Δ*fliC*	Fleiszig et al., 2001
PAO1-052	PAO1 Δ*pilA*	de Kerchove and Elimelech, 2007
PA14	Burn wound	Rahme et al., 1995
PA14 Δ*pqsA-E*	PQS mutant	Lars Dietrich
***P. aeruginosa*** **non-clone C environmental isolates**		
ATCC14886	Soil	Römling et al., 1994
PT4	Lake	Römling et al., 1994
PT6	Stagnant water body	Römling et al., 1994
***P. aeruginosa*** **clone C environmental isolates**		
W5Aug28	Water isolate	Pirnay et al., 2005
SG31M	Water isolate	Römling et al., 1994
PT31M	Drinking water	Römling et al., 1994
SG29M	Drinking water	Römling et al., 1994
***P. aeruginosa*** **non-clone C clinical isolates**		
DSM1128	Ear infection	Römling et al., 1994
ATCC3348	Acute infection	Römling et al., 1994
ATCC 27853	Blood culture	American Type Culture Collection (ATCC)
***P. aeruginosa*** **clone C clinical isolates**		
8277	Urine isolate	Römling et al., 2005
B6470	Ear infection	Römling et al., 2005
8735	Peritoneal dialysis	Römling et al., 2005
***E. coli*** **K-12 derivatives**		
TOP10	Cloning and propagating plasmids	Invitrogen
HB101 pRK2013	Host strain for pRK2013 conjugation helper plasmid, Km^r^	Ditta et al., 1980
DH5α λpir pTNS2	λpir host strain for pTNS2 to aid chromosomal integration of mini-Tn*7* element; R6K ori; Ap^r^	Choi and Schweizer, 2006

### Mutant Construction and Chromosomal Tagging

Deletion mutants were constructed in *P. aeruginosa* SG17M, our representative strain of clone C (Römling et al., 1994, 2005; Lee et al., 2015). Briefly, up- and down-stream regions (amplified with primers as in Table S1) of the *ftsH* target genes were cloned into a derivative of the pEX18Tc vector (Hoang et al., 1998), pSG001, that harbors an FLP-excisable Gm cassette between the BamHI and SalI restriction sites (Lee et al., 2015) and introduced into *E. coli* TOP10 strain. Conjugants were selected on VBMM medium with Gm after three-parental mating with the recipient SG17M, the plasmid-bearing donor *E. coli* TOP10 and *E. coli* HB101 harboring the helper plasmid pRK2013 (Choi and Schweizer, 2005). Tc^s^/Gm^r^ double crossover conjugants leaving 73 and 69 bp of *ftsH1* and *ftsH2* ORFs, respectively, were single colony purified and the pFLP2 plasmid (Table 2) was used to excise the Gm cassette. Cure from pFLP2 was on VBMM medium without Gm supplemented with 5% sucrose (Choi and Schweizer, 2005) and the gene deletion was confirmed by PCR using primers outside the region of homologous recombination.

**Table 2:** Plasmids used in this study.

**Plasmid**	**Description**	**References**
pEX18Tc	Gene replacement vector; Tc^r^, *oriT, sacB^+^*	Hoang et al., 1998
pSG001	pEX18T with FRT-Gm^R^-FRT cassette cloned between BamHI and SalI restriction sites	Lee et al., 2015
pSG052	pSG001-Δ*ftsH1* to create *ftsH1* deletion. Fragments up- and downstream of *ftsH1* cloned between SphI/HindIII and SacI/KpnI, respectively	This study
pSG053	pSG001-Δ*ftsH2* to create *ftsH2* deletion. Fragments up- and downstream of *ftsH2* cloned between SacI/SmaI and SalI/PstI, respectively	This study
pSG054	pSG001-*ftsH1*-*myc*, construct for chromosomal Myc-tag insertion. Fragments up and downstream of the *ftsH1* stop codon were cloned between HindIII/SphI and SacI/EcoRI, respectively	This study
pSG055	pSG001-*ftsH2*-*3xFLAG*, construct for chromosomal 3xFLAG tag insertion. Fragments up and downstream of the stop codon were cloned between SacI/SmaI and SalI/PstI, respectively	This study
pSG056	pUC18T-mini-Tn*7*T-Gm with *araC*-*ftsH1*-*myc* cloned between ApaI/SacI	This study
pSG057	pUC18T-mini-Tn7T-Gm with *araC-ftsH2*-3xFLAG cloned between ApaI/XhoI	This study
pSG058	pJN105 with *ftsH1* cloned between *Nhe*I/*XbaI*I	This study
pSG059	pJN105 with *ftsH2* cloned between NheI/XbaI	This study
pSG060	pJN105 with *ftsH1*-*Strep* cloned between NheI/XbaI	This study
pSG061	pJN105 with *ftsH2*-*Strep* cloned between NheI/XbaI	This study
pSG062	pSG060 derivative with FtsH1_H416Y_	This study
pSG063	pSG061 derivative with FtsH2_H420Y_	This study
pSG064	pJN105 with *6xHis-rpoH* cloned between NheI/XbaI	This study
pSG065	pJN105 with *phzC-6xHis* cloned between NheI/XbaI	This study
pJN105	Broad-host range vector with L-arabinose inducible *araBAD* promoter; pBBR1ori, Gm^r^	Newman and Fuqua, 1999
pFLP2	Vector for excision of FRT marker cassette; expression of FLP recombinase driven by λ promoter; Tc^r^	Hoang et al., 1998
pRK2013	Helper plasmid for mobilization of non-self-transmissible plasmids with *oriT*, RK2 transfer genes cloned in a ColEl replicon vector, *oriV*, Km^r^	Ditta et al., 1980
pTNS2	Helper plasmid for mini-Tn*7* integration, *tnsABCD*; R6K ori; Ap^r^	Choi and Schweizer, 2006
pUC18T-mini-Tn7T-Gm	Tn*7*L/Tn*7*R for insertion into the Tn*7* site; *ori*, ColE1-derived ori; *oriT*, origin of conjugative transfer; T0T1, transcriptional terminators; Ap^r^; *FRT*-Gm^r^-*FRT*	Choi and Schweizer, 2006
pBK-miniTn7-*gfp2*	miniTn7-*gfp*-*Gm* delivery vector	Koch et al., 2001

In order to tag *ftsH1* and *ftsH2* on the chromosome with Myc or 3xFLAG tag, respectively, the fragments upstream of the *ftsH* stop codon with the respective tag and the fragment downstream of the stop codon were cloned flanking the Gm marker in pEX18Tc. Subsequent conjugation, selection and Gm excision were performed similarly as for the gene deletions mentioned above (Choi and Schweizer, 2005).

### Plasmid Construction

Plasmids used in this study are described in Table 2. L-arabinose inducible broad-host range vector pJN105 (Newman and Fuqua, 1999) was used to clone all genes amplified from SG17M. *FtsH1* and *ftsH2* were amplified using primers *ftsH1* pJN F/R and *ftsH2* pJN F/R, respectively (Table S1). The amplified fragments were then cloned between NheI/XbaI restriction sites of the multiple cloning site. To construct catalytically inactive FtsH protease variants for substrate trapping, *ftsH1* and *ftsH2* were amplified from SG17M with a C-terminal Strep-tag, digested and cloned between the same sites of pJN105. QuickChange® PCR was used to generate the amino acid substitution H416Y for *ftsH1* and H420Y for *ftsH2* disrupting the Zn^2+^ binding site (Westphal et al., 2012). Candidate substrates *rpoH, lpxC*, and *minD*, were cloned into the pJN105 vector with N-terminal 6xHis tag, while *phzC1, phzF, nuoCD*, and ATP synthase subunit alpha were cloned in the same vector with a C-terminal 6xHis tag.

### Single Copy Complementation

A single gene copy was inserted into the *att*Tn*7* site downstream of the conserved *glmS* gene in *P. aeruginosa* SG17M (Choi and Schweizer, 2006). In brief, *ftsH1* and *ftsH2* with the *araC* promoter was subcloned from pSG058 and pSG059, respectively (Table 2) into the pUC18T-mini-Tn7T-Gm vector using ApaI/SacI and HindIII/SpeI restriction sites. Conjugation was performed by four-parental mating with the donor strain *E. coli* harboring pUC18T-mini-Tn*7*T derivatives, *E. coli* HB101 pRK2013 and DH5α λpir pTNS2 into the Tn*7* site of recipient strains SG17M Δ*ftsH1* or SG17M Δ*ftsH2*. Conjugants were selected and purified on VBMM medium. Insertion into the Tn*7* site was confirmed by PCR with primers binding to *glmS* and specific for mini-Tn*7* (Table S1).

### Bioinformatic Analysis

Protein sequences FtsH1 (acc. no.: EWH24232.1) and FtsH2 (acc. no.: EWH27927.1) of *P. aeruginosa* SG17M were used as queries to search for FtsH1 and FtsH2 homologs in the NCBI databases by BLASTP using standard parameters. Proteins homologs over the entire length of the sequence, >93%, were considered. These database searches retrieved FtsH1/FtsH2 homologs with an identity/homology limit of 46/64%. Proteins were aligned using ClustalX2 (Thompson et al., 1997) using standard parameters. As an outgroup, the FtsH homolog of *Gluconobacter oxydans* was selected which showed 92% query cover for FtsH1 of *P. aeruginosa*. The aligned sequences were subjected to phylogenetic analysis using neighbor-joining (NJ) and maximum likelihood (ML) in MEGA7.0 (Kumar et al., 2016). The Poisson model was used as an amino acid replacement model. The robustness of the phylogenetic tree topologies was evaluated by bootstrap analysis with 1,000 replications. The 16S rRNA gene sequences of the type strains of species included in the FtsH tree were retrieved from the NCBI database and phylogenetic analysis using NJ and ML (shown) was performed in MEGA7.0 with essentially the same results.

### Microfluidics Flow Cell Assay

Biofilm formation was assessed under continuous flow in a microfluidic chamber (Crusz et al., 2012). Strains under investigation were tagged with GFP at the *att*Tn*7* site (Klausen et al., 2003). The GFP-tagged strains were adjusted to an OD_600_ = 0.01 in ABTrace medium supplemented with 0.3 mM glucose and an inoculum of 300 μl was injected into each chamber. Biofilm formation was assessed at 37 and 21°C using Confocal Scanning Laser Microscopy (CSLM). Tobramycin (25 μg ml^−1^) was added for 20 h to the mature biofilm structure formed after 4 days at 37°C and 7 days at 21°C before image acquisition. Visualization of dead cells was done by staining with 0.3 μM propidium iodide.

### Pull-Down Assay for FtsH Substrates

The trap proteins FtsH1_H416Y_-Strep and FtsH2_H420Y_-Strep were expressed with 0.05% L-arabinose in SG17M Δ*ftsH1* and SG17M Δ*ftsH2*, respectively, and grown in 200 ml LB broth to an OD_600_ = 1 at 37°C. To cross-link, cells were washed with phosphate-buffered saline (PBS) and incubated at 37°C with 1% formaldehyde for 10 min. Cross-linking was quenched with 1M ice-cold glycine, cells were washed once with buffer W (100 mM Tris/HCl, pH 8.0, 150 mM NaCl, 1 mM EDTA) (IBA GmbH, Göttingen, Germany) and sonicated in 2 ml buffer W under cold conditions. The clear lysate was passed through an equilibrated *Strep*-Tactin® column (IBA GmbH, Göttingen, Germany). The bait variant protein with the trapped substrates was eluted using 0.5 column bed volume (CV) buffer E (Desthiobiotin) (IBA GmbH, Göttingen, Germany). Cross-linking was reversed by boiling for 20 min and the eluate was loaded onto SDS-PAGE (4% stacking/12% separating gel). Bands of interest were compared to a non-cross-linked and a bead control sample. Distinct protein bands reproducibly recovered on gels after cross-linking experiments were cut off the gel and subjected to LC-MS/MS.

### Hetero-Oligomer Formation

Formation of hetero-oligomers between FtsH1 and FtsH2 was tested by cross-linking the gene products of SG17M *ftsH1-*Myc and SG17M *ftsH2-*3xFLAG grown in LB at 37°C with *ftsH1-*Strep and *ftsH2-*Strep expressed from pJN105 with 0.05% L-arabinose added at OD_600_ = 0.4. Cells were harvested at OD_600_ = 0.7–0.8 and FtsH protein-protein interactions were stabilized by the addition of 1% formaldehyde for 10 min. Quenching, sonication, Strep-tag column purification, washing, elution, and reversal of the cross-linking were done similarly as for the pull-down assay. Plasmid encoded Strep-tagged proteins and chromosomally encoded Myc or 3xFLAG tagged proteins were detected by Western blotting.

### *In vivo* Degradation Assay

To assess protein degradation, N- and C- terminally 6xHis tagged genes in pJN105 were expressed in the SG17M wild type, the single *ftsH* deletion and the double *ftsH* deletion backgrounds. Cells grown overnight were diluted 1:100 in LB broth at 37°C until OD_600_ = 0.8. Expression of *rpoH* was induced by 1% L-arabinose, while expression of *phzC* was induced by 0.1% L-arabinose. After induction for 30 min at 37°C, protein translation was inhibited with 300 μg ml^−1^ spectinomycin. Samples were collected at different time points after blocking protein translation. Subsequently, the protein content in extracts was normalized by SDS-PAGE, equal amounts of protein loaded and target protein monitored by Western blotting using a 6xHis-tag antibody (Abcam).

### Cell Autolysis and Colony Morphology

A single colony was grown in tryptone broth overnight at 37°C. Ten microliters of OD_600_ = 1 suspension was spotted on Congo Red (CR) agar (Römling et al., 1998). The plates were incubated at 21, 28, and 37°C for 5, 2 and 1 day(s), respectively.

### Pyoverdine Secretion

*P. aeruginosa* secretes the two major siderophores pyoverdine and pyochelin with different spectral properties. Pyoverdine has an excitation/emission maximum at 400/447 nm, while pyochelin has the maxima at 313/430 nm (Folschweiller et al., 2002). To test pyoverdine production, a single colony was grown in tryptone broth overnight at 37°C. Ten microliters of OD_600_ = 1 was spotted on LB agar. The plate was incubated at 21, 28, and 37°C for 2, 2, and 1 day(s), respectively. Detection of pyoverdine secretion was performed with a handheld ultraviolet lamp (UVP, USA) of wavelength 365 nm.

### Transmission Electron Microscopy (TEM)

Bacterial cells in pellet were fixed by adding 2% glutaraldehyde in 0.2 M cacodylate buffer pH 7.4; rinsed in cacodylate buffer, embedded in 3% agarose and post-fixed for 2 h in 1% osmium tetroxide. After rinsing with H_2_O, samples were pre-stained over-night at 4°C in 0.5% aqueous uranyl acetate. Samples were then dehydrated and embedded in Spurr resin. Ultrathin sections were obtained with an ultramicrotome PT-PC PowerTome, RMC Boeckeler and grids were observed with a Hitachi HT7700 electron microscope.

### SDS-PAGE

After harvesting, equal aliquots of cells were resuspended in protein sample buffer, boiled for 15 min and loaded onto a SDS-PAGE gel with a 4% stacking and 10% or 12% separating gel. The protein content was normalized after visual inspection and samples were re-run on a SDS-PAGE gel. Protein bands were blotted onto a polyvinylidene difluoride membrane (Millipore) for 1 h.

### Western Blot

For detection of Myc- and 3xFLAG-tagged proteins, the membrane was blocked for 1 h in blocking solution (1x Tris-buffered saline (TBS) pH 7.4, 0.01% Tween 20, 5% skim milk powder). Proteins were detected with 1:2,000 anti-Myc (Thermo Scientific) and anti-3xFLAG (Sigma) antibody overnight. The secondary antibody was 1:3,000 diluted peroxidase-conjugated goat anti-mouse IgG antibody (Jackson Immuno Research, UK). For detection of T3SS effector proteins, the membrane was incubated overnight with antisera against ExoS, ExoT, and ExoY diluted 1:2,000 in 5% skimmed milk. Primary antibodies were detected with peroxidase-conjugated goat anti-rabbit IgG antibody (Jackson Immuno Research, UK) in 5% skimmed milk.

For the detection of 6xHis-tagged proteins, the membrane was blocked with 5% Bovine Serum Albumin (BSA) (Roche). Proteins were detected with 1:2,000 anti-penta His-tag HRP conjugate (Qiagen).

Enhanced chemiluminescence (ECL) detection reagent (Roche) was used to monitor horseradish peroxidase activity by chemoluminescence through the oxidation of luminol.

## Data Availability

All datasets generated for this study are included in the manuscript and/or the Supplementary Files.

## Author Contributions

UR conceived the study and designed the research. UR, SK, CL, RB, MR, and TT-N designed the experiments. SK, CL, MR, TT-N, MN, SS, JD, RB, CG, JT, LD, LJ, and UR performed the research. SK, CL, MR, JD, RB, MN, TT-N, and UR analyzed the data. SK and UR wrote the paper. All authors revised the manuscript and commented on the final version.

### Conflict of Interest Statement

The authors declare that the research was conducted in the absence of any commercial or financial relationships that could be construed as a potential conflict of interest.
