# Supplementary material for: Two FtsH Proteases Contribute to Fitness and Adaptation of Pseudomonas aeruginosa Clone C Strains
Source: Front Microbiol. 2019 Jul 9;10:1372. doi: 10.3389/fmicb.2019.01372 (PMC6629908; doi:10.3389/fmicb.2019.01372)
Supplement: Supplementary file 3 [file Data_Sheet_1.pdf]

Supplementary Material

Two FtsH proteases contribute to fitness and adaptation of *Pseudomonas aeruginosa* clone C strains

Shady Mansour Kamal[1,2], Morten Levin Rybtke[3], Manfred Nimtz[4], Stefanie Sperlein[1], Christian Giske[5], Janja Trček[6], Julien Deschamps[7], Romain Briandet[7], Luciana Dini[8], Lothar Jänsch[4], Tim Tolker-Nielsen[3], Changhan Lee[1†] and Ute Römling[1*]

[1] Department of Microbiology, Tumor and Cell Biology, Karolinska Institutet, Stockholm, Sweden
[2] Department of Microbiology and Immunology, Faculty of Pharmaceutical Sciences & Pharmaceutical Industries, Future University in Egypt, New Cairo, Egypt
[3] Department of Immunology and Microbiology, Faculty of Health and Medical Sciences, University of Copenhagen, Copenhagen, Denmark
[4] Cellular Proteomics, The Helmholtz Centre for Infection Research, Braunschweig, Germany
[5]Division of Clinical Microbiology, Department of Laboratory Medicine, Karolinska Institutet, Stockholm, Sweden
[6] Department of Biology, Faculty of Natural Sciences and Mathematics, University of Maribor, Maribor, Slovenia
[7]Micalis Institute, INRA, AgroParisTech, Université Paris-Saclay, Jouy-en-Josas, France
[8]Department of Biological and Environmental Sciences and Technologies (DiSTeBA), University of Salento, Lecce, Italy

[†]**Current affiliation:**
Department of Molecular, Cellular and Developmental Biology, University of Michigan, Ann Arbor, USA

[*]**Correspondence:**
Ute Römling
Ute.Romling@ki.se

SUPPLEMENTARY EXPERIMENTAL PROCEDURES

Bacterial Growth Assessment in Liquid Culture

To assess the growth rate, a single colony was inoculated in LB broth (BD Difco) and incubated at 37°C overnight with shaking at 200 rpm. The overnight culture was diluted to OD_{600} = 0.02 in LB and M63-citrate minimal medium. In a honeycomb 100-well plate (Bioscreen, Cat no. 9502550X), 300 µl of the cell suspension was loaded into wells with a minimum of 6 replicates for each strain, at 37°C shaking incubation using BioScreen C automatic fermentor (Oy Growth Curves AB Ltd, Helsinki, Finland). The filter used to measure OD was 420-600 nm Wideband.

Bacterial Growth Assessment on Solid Medium

Growth was also assessed by the spot assay on LB agar at 37°C. After overnight growth, a single colony was inoculated in LB broth and incubated overnight at 37°C in a 200 rpm shaking incubator. The OD_{600} was adjusted to one and the culture was 10-fold diluted until 10^{-6}. Five µl of each dilution was spotted on LB agar medium, and incubated at 37°C.

Virulence Assay in *Galleria mellonella*

One ml of cells grown overnight in LB at 37°C was washed with one ml Phosphate-Buffered Saline (PBS) and adjusted to OD_{600} = 2. The cell suspension was serially diluted and ten µl of a 10^{-5} dilution (corresponding to around 60 CFU) was injected into the last left leg of the larvae (Livefood UK-5060446440117) using a syringe (VWR 720-2554, insulin injection) (Pustelny et al., 2013). Per strain, 80 larvae were injected and incubated in the dark at 30°C. Non-injected and PBS injected larvae were included as handling control. Viability was recorded every 12 h by assessing larval death judged by complete lack of movement upon touching and turning.

Type III Secretion System (T3SS) Assay

The cell suspension grown overnight at 37°C in modified LB medium containing 200 mM NaCl, 10 mM $MgCl_2$ and 0.5 mM $CaCl_2$ (LB-MC) was diluted 1:300 and grown to mid log phase at 37°C in LB-MC containing 5 mM EGTA to remove calcium ions from the medium (Toska et al., 2014). Effector proteins were precipitated from two ml supernatant by 10% trichloroacetic acid (final concentration) and pelleted by centrifugation. Proteins were washed with cold acetone, dried by evaporation and resuspended in 1x SDS sample buffer. Aliquots of protein samples normalized to OD_{600} = 1 for each culture were applied.

Motility Assays

Flagella-dependent swimming motility was assessed by inoculation of a single colony (grown on LB agar overnight at 37°C) halfway into a swimming agar plate (1% tryptone, 0.5% NaCl, 0.3% agar) (Rashid and Kornberg, 2000). The plate was incubated at 37°C for 18-22 h, and the diameter of the swimming zone was measured. Type IV-pili-dependent twitching motility was assessed by stab-inoculating a single

colony (grown on LB agar overnight at 37°C) to the bottom of a polystyrene petri dish plate covered by twitching agar medium (1% tryptone, 0.5% NaCl, 0.5% yeast extract, 0.3% agar) (Darzins, 1993). After incubation at 37°C for 21 h, the diameter of the twitching zones was measured. To aid visualization of the twitching zones, the plate was flooded with twitching motility developer solution (TEM) (glacial acetic acid: methanol: water, 1:5:4 ratio) and incubated for 30 min.

Biofilm Formation in Steady State Cultures

To assess biofilm formation, 200 µl of an overnight culture grown in LB, adjusted to OD_{600} = 0.01 was loaded into a 96-well polystyrene plate with a flat bottom (*TPP*, Switzerland) and incubated at 37°C for 24 h in a moist chamber. Wells were washed and stained with 250 µl 0.4% crystal violet (CV). The stained biofilm formed on the wall of the well dissolved in 300 µl 30% acetic acid and the OD_{600} was measured (O'Toole, 2011). The OD_{600} absorbance values were normalized to the OD_{600} growth values.

To visualize biofilm formation, 250 µl of an overnight culture grown in tryptone soy broth (TSB) at 30°C adjusted to OD_{600} = 0.01 was loaded into a 96-well plate (Greiner Bio One, µclear, 655090) and incubated 1 h at 30°C for initial adhesion. Biofilms were allowed to develop for 24 h after 250 µl fresh TSB was added. After addition of 50 µl of Syto9/propidium iodide live/dead stain, cells and eDNA were analyzed in the biofilm under a Leica SP2 confocal laser scanning microscope at the INRA-Mima2 imaging platform. 3D projections from z-series constructed by Imaris (Bitplan) with structural parameters extracted using the PHILIP Matlab routine. Three independent experiments were performed. Principal component analysis was performed using the extracted geometric biofilm parameters biovolume, thickness and roughness.

Antimicrobial Susceptibility Test

Inoculum preparation and antibiotic disk diffusion test with antibiotics including the clinically relevant aminoglycosides gentamicin (10 µg per disk) and tobramycin (10 µg per disk) was done according to the guidelines of EUCAST (Matuschek et al., 2014). The inoculum of the bacterial suspension was adjusted to McFarland 0.5 in saline and spread on a surface of Mueller Hinton agar using a cotton swab. Antibiotics discs were applied and the plates were incubated at 35°C for 20 h.

***Pseudomonas* Quinolone Signal (PQS) Secretion Assay**

A single colony was inoculated in 25 ml LB medium in 250 ml flask and incubated for 20 h at 37°C. OD_{600} was adjusted to one in 5 ml LB. Supernatant was collected and filtered through 0.2 µM cellulose acetate filter (VWR). PQS in the supernatant was extracted with five ml ethyl acetate acidified with 0.01% (v/v) glacial acetic acid (Fletcher et al., 2007). After drying the organic layer, the pellet was resuspended in 300 µl methanol, evaporated and resuspended in 50 µl methanol. Normal phase silica 60F254 thin layer chromatography (TLC) plates were activated by soaking in 5% (w/v) KH_2PO_4 for 30 min followed by incubation at 70°C for 1 h. Eight µl of the

extracted PQS were loaded on the activated TLC plate and run for 30-45 min in dichloromethane-methanol mixture (95:5 v/v). The bands were detected under UV light of 312 nm.

Heat Shock Tolerance Assay

P. aeruginosa cells incubated in LB broth with shaking at 200 rpm at 37°C were harvested after 16 h of growth. OD_{600} was adjusted to one and 500 µl of cell suspension was incubated at 50°C for 30 and 60 min while a control cell suspension was kept on ice. Cell viability was assessed by the spot assay with ten µl of serially diluted cells on LB agar and incubated at 37°C for 12-14 h of incubation.

Hypochlorous Acid (HOCl) Stress Assay

HOCl stock solution was freshly prepared by diluting concentrated sodium hypochlorite (NaOCl-10-15% active chloride) 1:1000 volume ratio with 40 mM potassium phosphate buffer (KPi), pH 7.5 (Groitl et al., 2017). The HOCl stock solution was serially diluted in 10 mM NaOH and the concentration was determined using the extinction coefficient A_{292nm} 350 $M^{-1}cm^{-1}$ (Morris, 1966). Cells were harvested from stationary phase after growth in M63 citrate for 18 h at 37°C, 200 rpm. 2 ml cell suspension adjusted to $OD_{600} = 0.5$ was resuspended in 2 ml pre-warmed M63-citrate medium containing 20 µM HOCl and incubated at 37°C for 30 min. A control sample without HOCl treatment was included. Cells were washed with one ml quench solution (M63 medium containing 10 mM $Na_2S_2O_3$). Cell viability was assessed by serial dilution in 0.85% NaCl. Five µl of each dilution was spotted on LB agar and incubated at 37°C for 12-14 h.

SUPPLEMENTARY FIGURES

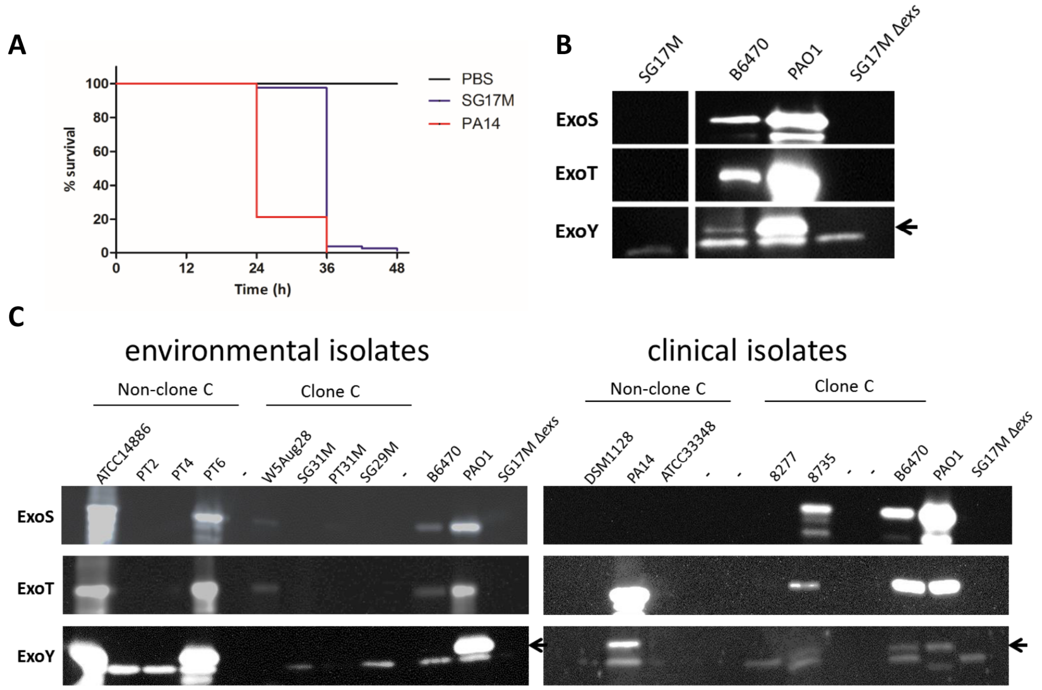

Figure S1. Virulence characteristics of the environmental isolate *P. aeruginosa* SG17M. **(A)** Kaplan–Meier survival curves for *Galleria mellonella* larvae to assess the virulence of *P. aeruginosa* SG17M and the highly virulent reference strain *P. aeruginosa* PA14 in this non-mammalian host. PBS injection was the technical control and survival of larvae was monitored every 12 h. Secretion of type three secretion system (T3SS) effector proteins in *P. aeruginosa* SG17M **(B)** and a panel of *P. aeruginosa* clone C and non-clone C strains of environmental and clinical origin (**C**; see table 1). Antibodies against ExoS, ExoT and ExoY were used to detect secreted proteins in the supernatants of cells grown to logarithmic phase at 37°C. The position of the ExoY signal is indicated by an arrow. *P. aeruginosa* PAO1, PA14 and clone C strain B6470 were used as positive controls. No signals are expected for DSM1128 as it belongs to the *P. aeruginosa* PA7 taxonomic outlier strains that do not encode T3SS. *P. aeruginosa* SG17M Δ*exs* is the negative control. The panel in B is derived from one blot.

A

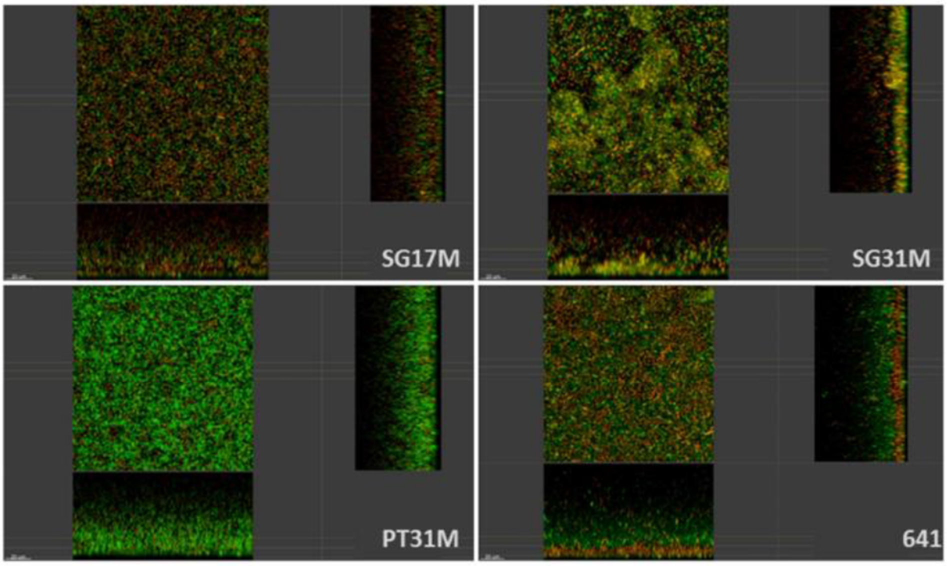

B

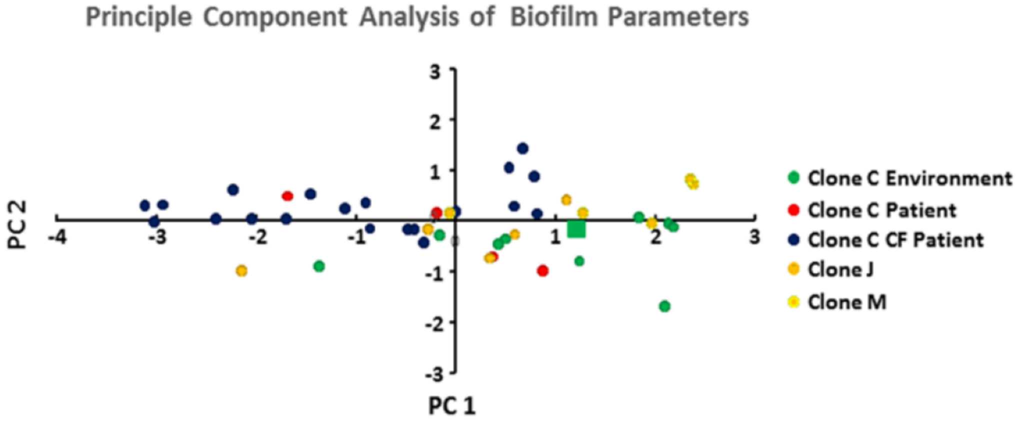

Figure S2. Comparative characteristics of steady-state biofilms of *P. aeruginosa* SG17M with selected clinical and environmental isolates of clone C and non-clone C origin. **(A)** Biofilm formation of SG17M is characterized by a voluminous unstructured biofilm with a smooth surface. A relatively high percentage of dead cells is observed. Natural water line strain SG31M and technical water line strains PT31M and 641 are shown for comparison. **(B)** Principal component analysis of environmental, clinical and cystic fibrosis clone C strains compared to clone J and M isolates using biofilm parameters biovolume, thickness and roughness. SG17M data is indicated by a cube. There is no clustering according to clonality, however, environmental isolates form a loose cluster.

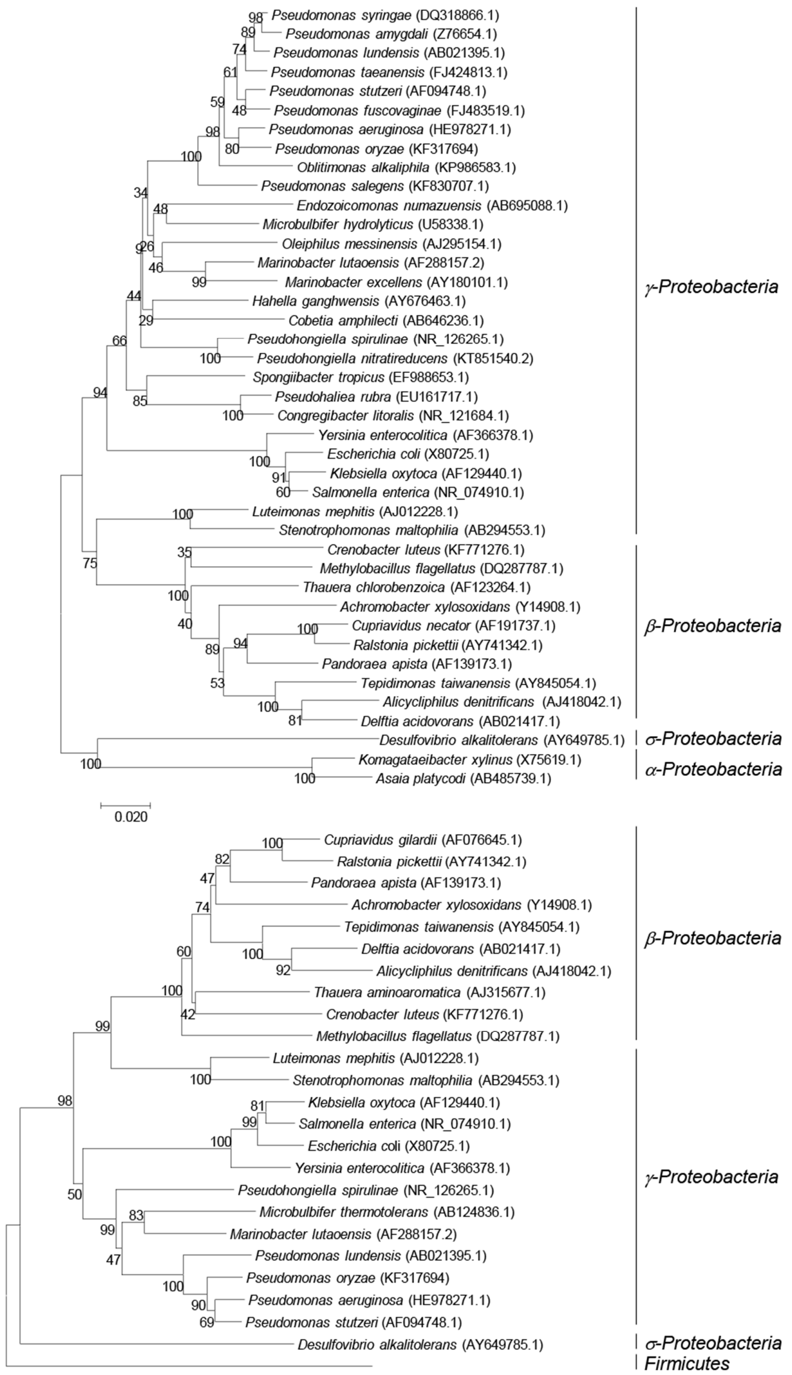

Figure S3. Phylogenetic analysis of corresponding 16S rRNA gene sequences of relevant FtsH1 (upper tree)/FtsH2 (lower tree) protein encoding bacterial species. Examples of strains with multiple copies (including at least one FtsH2 homolog) are: *Ralstonia pickettii* DTP0602 (3 homologs), *Alicycliphilus denitrificans* K601 (2 homologs), *Pseudomonas lundensis* L1819 (2 homologs) and *Streptococcus pneumoniae* SMRU2535 (2 homologs). *Gluconobacter oxydans* is included as an outgroup. Branch lengths correspond to substitutions per site, bootstrap values are indicated in %. The sequence accession numbers are shown in parenthesis.

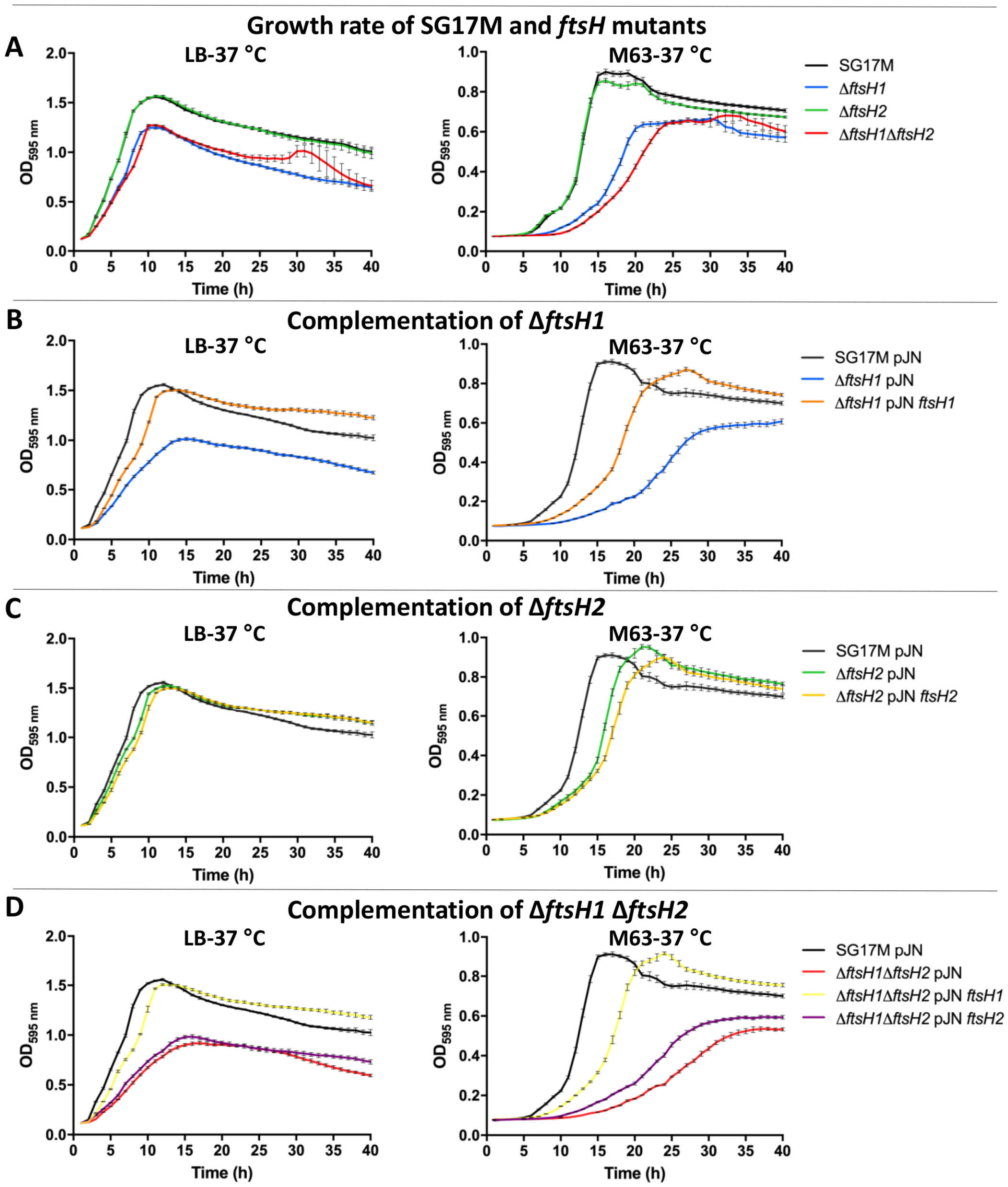

Figure S4. FtsH1 and FtsH2 contribute to growth in rich and minimal medium at 37°C. Assessment of growth rate in rich LB medium (left panel) and minimal medium M63 (right panel) using Bioscreen C machine taking automated measurements every hour **(A)**. Complementation of *ftsH1* single deletion mutant **(B)**, *ftsH2* single deletion mutant **(C)** and *ftsH1 ftsH2* double deletion mutant **(D)** at 37°C in LB (left panel) and M63 medium (right panel). Induction with L-arabinose is not required for optimal complementation. One representative experiment of two independent biological replicates with congruent results is shown. Error bars indicate SD for 16 technical replicates (A) and 8 technical replicates (B, C and D). Note that in M63 medium, the vector control in the *ftsH2* background causes growth retardation compared to the wild type (compare A with C).

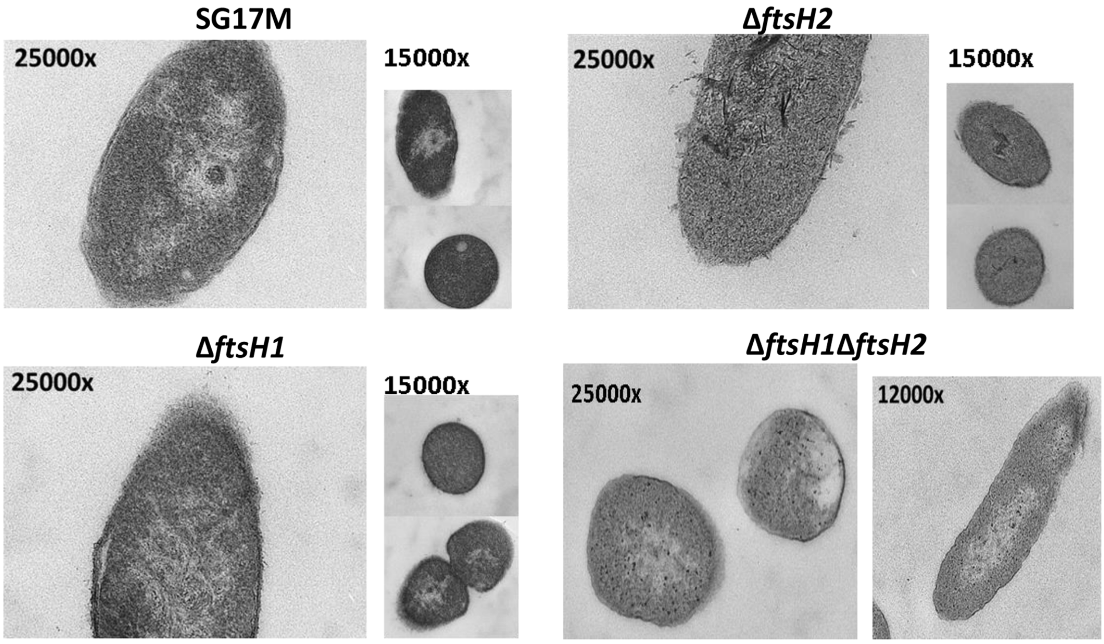

Figure S5. Transmission electron microscopy of *P. aeruginosa* SG17M and *ftsH* mutants shows no difference in cell morphology. Strains were grown in LB medium at 37°C to $OD_{600} = 0.9$.

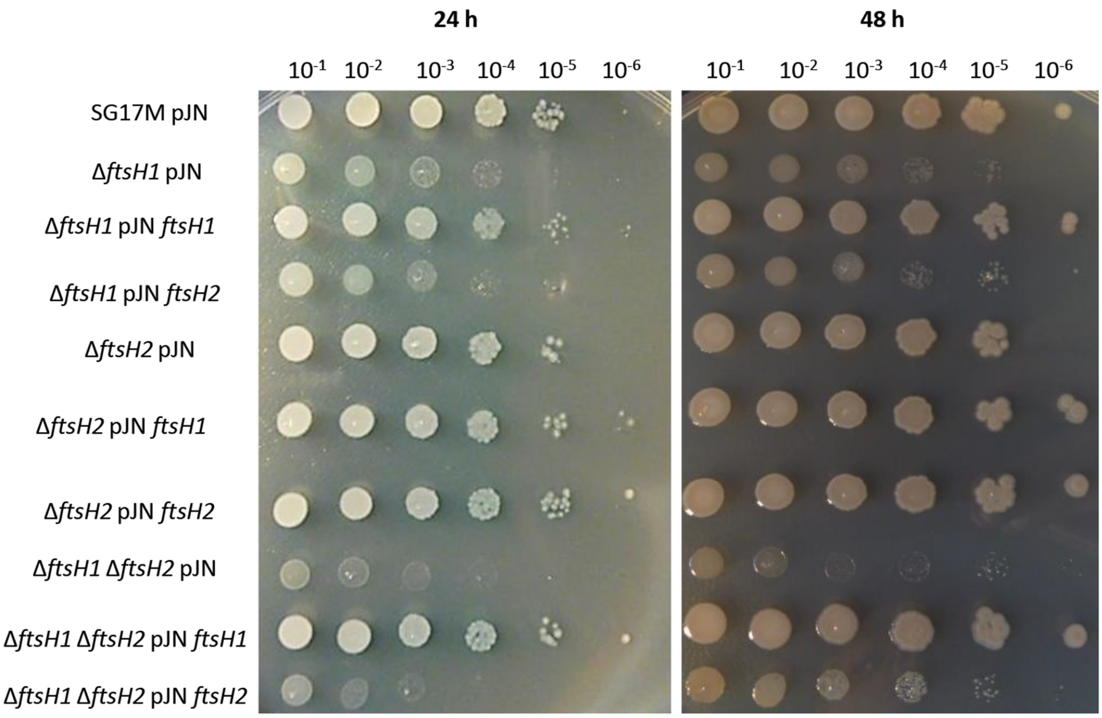

Figure S6. Spot assay to test complementation of growth on solid LB agar using the expression vector pJN105 (pJN) at 37°C. The smaller colony size at equal cell number indicates growth retardation of the *ftsH1* mutant and the *ftsH1 ftsH2* double mutant. pJN*ftsH1* = *ftsH1* cloned in pJN105. pJN*ftsH2* = *ftsH2* cloned in pJN105.

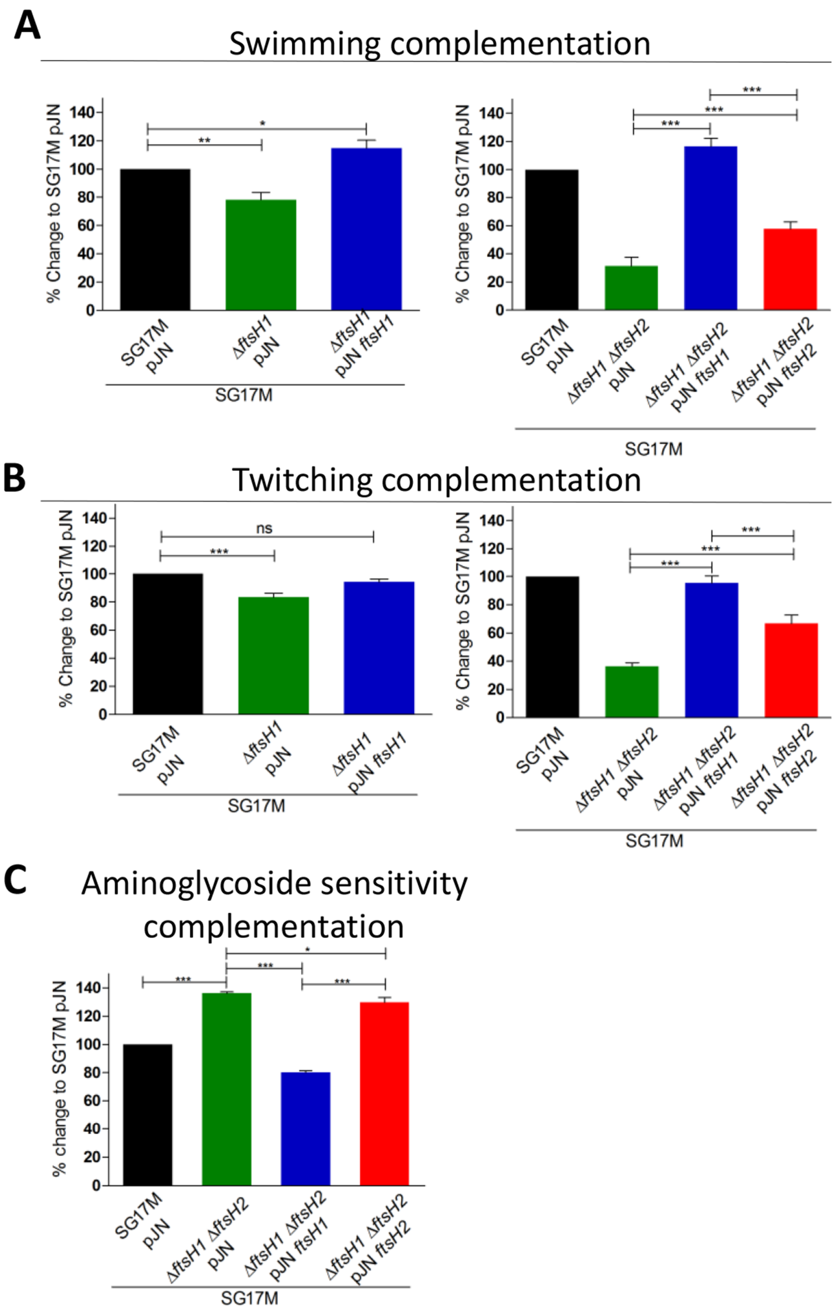

Figure S7. Complementation of swimming and twitching motility and antimicrobial resistance phenotypes of *P. aeruginosa* SG17M *ftsH* mutants. Flagella-dependent swimming motility **(A)** and type IV pili-dependent twitching motility **(B)** were complemented at 37°C by overexpression of *ftsH1* and *ftsH2* from vector pJN105 (pJN). The mean value was calculated from 6 technical replicates of two independent experiments. Error bars indicate standard deviation SD (*** *P*<0.0001). **(C)** Complementation of the sensitivity phenotype against the aminoglycoside tobramycin in the *P. aeruginosa* SG17M Δ*ftsH1* Δ*ftsH2* double mutant at 37°C by overexpression of *ftsH1* and *ftsH2* from vector pJN105 (pJN). pJN*ftsH1* = *ftsH1* cloned in pJN105. pJN*ftsH2* = *ftsH2* cloned in pJN105. The mean value was calculated from 6 technical replicates of two independent experiments. Error bars indicate SD (*** *P*<0.0001).

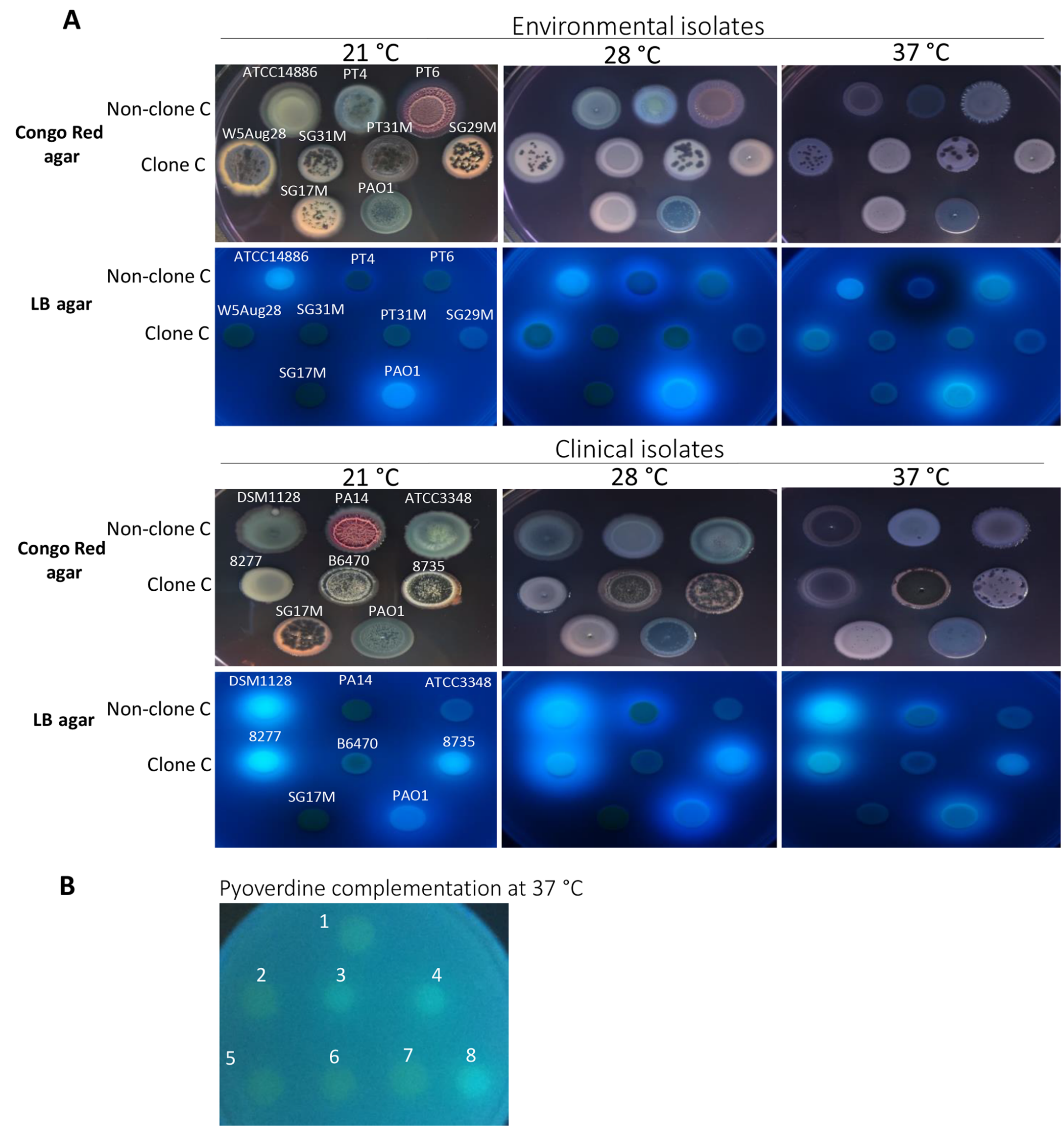

Figure S8. Panel of *P. aeruginosa* clone C and non-clone C strains of environmental and clinical origin shows a variety of phenotypes. **(A)** Colony morphology (autolysis; upper panel) was tested on Congo Red agar at 21, 28 and 37°C. Colonies were documented after five days at 21°C, two days at 28°C and one day at 37°C. *P. aeruginosa* SG17M and PAO1 were reference strains. Pyoverdine secretion (lower panel) was assessed on LB agar at 21, 28 and 37°C illuminated with UV at 365 nm. Pyoverdine production was documented after two days at 21 and 28°C and after one day at 37°C. *P. aeruginosa* PAO1 and SG17M were a positive and a negative control, respectively. **(B)** Complementation of pyoverdine secretion at 37°C. Numbers correspond to the following strains: 1, SG17M pJN105; 2, Δ*ftsH2* pJN105; 3, Δ*ftsH1* pJN105; 4, Δ*ftsH1* Δ*ftsH2* pJN105; 5, Δ*ftsH2* p*ftsH2*; 6, Δ*ftsH1* p*ftsH1*; 7, Δ*ftsH1* Δ*ftsH2* p*ftsH1*; 8, Δ*ftsH1* Δ*ftsH2* p*ftsH2*. p*ftsH1* = *ftsH1* cloned in plasmid pJN105; p*ftsH2* = *ftsH2* cloned in plasmid pJN105.

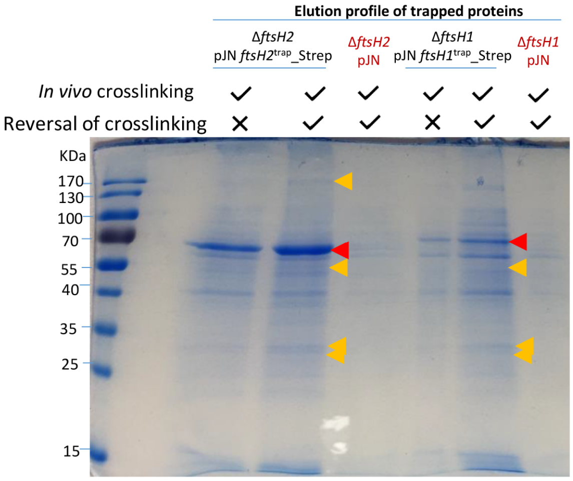

Figure S9. SDS-PAGE gel displaying potential interaction partners of FtsH1^{trap} and FtsH2^{trap}. Trapped proteins were cross-linked with bait proteins FtsH1^{trap} and FtsH2^{trap} in the respective deletion mutant backgrounds *in vivo* and pulled down with biotin beads after cell breakage. After resuspension in sample buffer, the samples were applied to the SDS page gel without (cross-linking not reversed) and with (cross-linking reserved) boiling. Extracts derived from *ftsH1* and *ftsH2* single deletion mutants with an empty vector were the matrix control. Bait proteins FtsH1^{trap} and FtsH2^{trap} are marked with red arrows, whereas the yellow arrows indicate the protein bands containing trapped proteins that were cut out of the gel for further analysis.

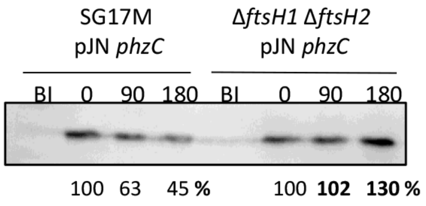

Figure S10. Degradation of PhzC-6xHis is *ftsH1* dependent in *P. aeruginosa* SG17M. *In vivo* degradation assay in the logarithmic growth phase at 37°C. BI (Before Induction) refers to the sample before inducing the expression of *phzC*. Numbers above the blot represent time in min after the inhibition of translation with 300 µg ml^{-1} spectinomycin, which was preceeded by 30 min induction with 0.1% L-arabinose. The intensity of the PhzC-6xHis signal at time 0 is set to 100%. The band intensities after 10 sec exposure time were quantified using the software ImageJ.

SUPPLEMENTARY TABLES

Table S1. Primers used in this study

Primer name	Sequence[1,2] (5'-3')	Purpose	Source or reference
ftsH1 Up SphI F	CGGCATGCGATGATCAGCCACAGAATCAGATT	Construction of Δ*ftsH1* (Upstream region)	This study
ftsH1 Up HindIII R	CGCAAGCTTTGGTGACCGATAGATTTGAACTG	Construction of Δ*ftsH1* (Upstream region)	This study
ftsH1 Dn SacI F	CGCGGAGCTCAGCCCAGCTTGAGAACGAAGT	Construction of Δ*ftsH1* (downstream region)	This study
ftsH1 Dn kpnI R	CGGGTACCAGAACACCCCTCCCATCGGT	Construction of Δ*ftsH1* (downstream region)	This study
ftsH2 Up SacI F	CGCGAGCTCTTCAACCCGACGTTGATTCC	Construction of Δ*ftsH2* (Upstream region)	This study
ftsH2 Up XmaI R	CGCCCGGGCGACGATCCAGTAGCCAATG	Construction of Δ*ftsH2* (Upstream region)	This study
ftsH2 Dn SalI F	CCGGTCGACCTCTCTACACCCCAAGGGTCC	Construction of Δ*ftsH2* (downstream region)	This study
ftsH2 Dn PstI R	CGCTGCAGTCCCAGCCACATCAACATCA	Construction of Δ*ftsH2* (downstream region)	This study
GM 3'check F	TTTGATATCGACCCAAGTACCG	Confirming upstream region cloning in pSG052 and pSG053	This study
GM 5' check R	AATTCGATCCATTGCTGTTGAC	Confirming downstream region cloning in pSG052 and pSG053	This study
ftsH1 b4DKO check F	AGGCCGACTCGAACATGTAAC	Confirmation of Δ*ftsH1*	This study
ftsH1 b4UKO check R	GCTACGATCAATACCGGTTTCA	Confirmation of Δ*ftsH1*	This study
ftsH2 b4UKO check F	CGTACATCTTCGACAGCCAA	Confirmation of Δ*ftsH2*	This study
ftsH2 b4DKO check R	AACACTTCGAGCAGCACG	Confirmation of Δ*ftsH2*	This study
*ftsH1*Myc Up HindIII F	CGCAAGCTTATCGTCGACATGCGCGAGTTC	Chromosomal insertion of Myc tag at the C-terminal end of *ftsH1*	This study
ftsH1 Myc Up SphI R	CGGCATGCTCA**CAGGTCCTCCTCGCTGATGAGCTTCTGCTC**GTGCTCGCCGGCCGGCCCACCGAT	Chromosomal insertion of Myc tag at the C-terminal end of *ftsH1*	This study
ftsH1 Myc Dn SacI F	CGCGGAGCTCGAGCCCTGAATGAGCAATC	Chromosomal insertion of Myc-tag at the C-terminal end of *ftsH1*	This study
ftsH1 Myc Dn EcoRI R	CGCCGGAATTCCCACGTCATGCACTCGAATTA	Chromosomal insertion of Myc-tag at the C-terminal end of *ftsH1*	This study
ftsH2 Flag Up SacI F	CGCGAGCTCAAGAAGGGACGGCTGGACATC	Chromosomal insertion of 3xFLAG at the C-terminal end of *ftsH2*	This study
ftsH2 Flag Up XmaI R	CGCCCGGGTCA**CTTGTCGTCGTCGTCCTTGTAGTCGATGTCGTGGTCCTTGTAGTCGCCGTCGTGGTCCTTGTAGTC**TGGTGTGGACCCTTGGGGT	Chromosomal insertion of 3xFLAG at the C-terminal end of *ftsH2*	This study
ftsH2 Flag Dn SalI F	CCGGTCGACGCGAACACCTTCACCTCGTTTG	Chromosomal insertion of 3xFLAG at the C-terminal end of *ftsH2*	This study
ftsH2 Flag Dn PstI R	CG§TGTCCCAGCCACATCAACATCA	Chromosomal insertion of 3xFLAG at the C-terminal end of *ftsH2*	This study
araC ftsH2 Tn7 *Hind*III F	CGCAAGCTTTGCATAATGTGCCTGTCAAATGG	*ftsH2* insertion into Tn*7* site	This study
araC ftsH2 mini-Tn7 *Spe*I R	GCGACTAGTTCATGGTGTGGACCCTTGG	*ftsH2* insertion into Tn*7* site	This study
ftsH1 pJN NheI F	CGCGCTAGCGCCGGCTTCAGAGGGTAG	Cloning *ftsH1* for expression	This study
ftsH1 pJN XbaI R	GCGTCTAGACTCATTCAGGGCTCTCAGTGC	Cloning *ftsH1* for expression	This study

ftsH2 pJN Nhe F	CGCGCTAGCCGACTTGCGAGGAACATTGC	Cloning *ftsH2* for expression	This study
ftsH2 pJN XbaI R	GCGTCTAGATCATGGTGTGGACCCTTGG	Cloning *ftsH2* for expression	This study
ftsH1 Strep pJN XbaI R	GCGTCTAGATCA**TTTTTCGAACTGCGGGTGGCTCCA**GTGCTCGCCGGCCGGCCCACCGAT	Cloning FtsH1-Strep for trap generation	This study
ftsH2 Strep pJN XbaI R	GCGTCTAGATCA**TTTTTCGAACTGCGGGTGGCTCCA**TGGTGTGGACCCTTGGGGT	Cloning FtsH2-Strep for trap generation	This study
ftsH1 Strep H416Y F	GCGCAACACCGCCTACTACGAAGCCGGCCACGC	Amino acid mutagenesis FtsH1$_{H416Y}$	This study
ftsH1 Strep H416Y R	GCGTGGCCGGCTTCGTAGTAGGCGGTGTTGCGC	Amino acid mutagenesis FtsH1$_{H416Y}$	This study
ftsH2 Strep H420Y F	GGGAAACCGTGGCCTATTACGAGATGGGCCATGCG	Amino acid mutagenesis FtsH2$_{H420Y}$	This study
ftsH2 Strep H420Y R	CGCATGGCCCATCTCGTAATAGGCCACGGTTTCCC	Amino acid mutagenesis FtsH2$_{H420Y}$	This study
pJN MCS R	AAACGACGGCCAGTGAGC	Confirmation of cloning in pJN expression vector	This study
pjN_pBAD F	CCATAGCATTTTTATCCATAAG	Confirmation of cloning in pJN expression vector	(Lee et al., 2015)
PglmS-up	CTGTGCGACTGCTGGAGCTGA	Confirmation of genome integration	(Choi and Schweizer, 2006)
PglmS-down	GCACATCGGCGACGTGCTCTC	Confirmation of genome integration	(Choi and Schweizer, 2006)
Tn7_R	CACAGCATAACTGGACTGATTTC	Confirmation of genome integration	(Choi and Schweizer, 2006)
Tn7 L	ATTAGCTTACGACGCTACACCC	Confirmation of genome integration	(Choi and Schweizer, 2006)
RpoH NheI His F	CGCGCTAGCTCGTGAATCGGAGGATTCGCATG**CACCACCACCACCACCAC**CACTTCTTTGCAACCTGT	Cloning *rpoH*-6xHis in pJN105	This study
RpoH XbaI R	GCGTCTAGATCAGGCGAGAATCCGCCCTTTCA	Cloning *rpoH*-6xHis in pJN105	This study
PhzC NheI F	CGCGCTAGC GAAGGGGGACCCACCATGGA	Cloning *phzC* 6xHis in pJN105	This study
PhzC XbaI His R	GCGTCTAGATCA**GTGATGATGATGATGATG**TGCCACGGTCTCCAGGGGGAA	Cloning *phzC* 6xHis in pJN105	This study

[1] Restriction sites are underlined

[2] Tag sequences are indicated in bold

Table S2. List of interacting proteins pulled down by trap variants of FtsH1 and FtsH2 proteases

List of proteins[a] pulled down with FtsH1[trap]				
**Name**	**Accession Number (Uniprot)**	**Average Mass (Da)**	**Coverage/ peptides identified**	**Confirmation of interaction and Reference**
**Trigger factor**	Q9I2U2	48582	32%-13	associated with FtsH in *E. coli* (Arifuzzaman et al., 2006)
**Peptidyl-prolyl cis-trans isomerase**	P30417	26336	29%-6	-
**ATP synthase subunit alpha**	Q9HT18	55393	25%-13	a substrate for FtsH in *E. coli* (Akiyama et al., 1996)
**HflC**	Q9HUM3	33116	22%-7	associated with FtsH in *E. coli* (Kihara et al., 1996)
**ATP-dependent protease ATPase subunit HslU**	Q9HUC5	46101	16%-7	-
**Phzf**	C7AUN6	14087	16%-2	-
**Septum site-determing protein MinD**	J3GZN1	29218	8%-2	interacts with FtsZ/FtsA/FtsW in *E. coli* (Shen and Lutkenhaus, 2009)
**Phosphoglucosamine mutase GlmM**	Q9HV50	47751	7%-3	co-occurrence across genomes and coexpressed (Tavares et al., 2003)
**PhzC**	A0A0H2ZL76	44312	4%-2	a substrate (this study)
**FtsH2**	S1EWL6	63375	3%-2	associated (this study)

List of proteins[a] pulled down with FtsH2^{trap}				
**Name**	**Accession Number (Uniprot)**	**Average Mass (Da)**	**Coverage/ peptides identified**	**Confirmation of interaction and Reference**
**HflC**	Q9HUM3	33116	21%-7	associated with FtsH in *E. coli* (Kihara et al., 1996)
**Peptidyl-prolyl cis-trans isomerase**	F6AIP0	26336	13%-3	-
**ATP synthase subunit alpha**	L8MV38	55228	11%-6	a substrate for FtsH in *E. coli* (Akiyama et al., 1996)
**Aspartokinase**	I6GDZ5	16400	8%-1	-
**Signal peptidase I**	A6VAK8	32145	7%-1	-
**FtsH1**	Q9HV48	69955	6%-3	associated (this study)
**Putative oxidoreductase, aryl-alcohol dehydrogenase like protein**	K8GE23	38409	4%-2	-
**Acyltransferase**	L0D9Q2	44137	2%-2	-
**Glycerol-3-phosphate dehydrogenase**	Q9I3A8	53486	2%-1	-
**Probable protein kinase UbiB**	L8MJY1	60755	2%-1	-

[a] Proteins were selected based on the highest coverage/number of peptides detected by MS/MS for each of the protein bands.

REFERENCES

Akiyama, Y., Kihara, A., Tokuda, H., and Ito, K. (1996). FtsH (HflB) is an ATP-dependent protease selectively acting on SecY and some other membrane proteins. *J Biol Chem* 271, 31196-31201.

Arifuzzaman, M., Maeda, M., Itoh, A., Nishikata, K., Takita, C., Saito, R., et al. (2006). Large-scale identification of protein-protein interaction of *Escherichia coli* K-12. *Genome Res* 16, 686-691. doi: 10.1101/gr.4527806.

Choi, K.H., and Schweizer, H.P. (2006). mini-Tn7 insertion in bacteria with single *att*Tn*7* sites: example *Pseudomonas aeruginosa. Nat Protoc* 1, 153-161. doi: 10.1038/nprot.2006.24.

Darzins, A. (1993). The *pilG* gene product, required for *Pseudomonas aeruginosa* pilus production and twitching motility, is homologous to the enteric, single-domain response regulator CheY. *J Bacteriol* 175, 5934-5944.

Fletcher, M.P., Diggle, S.P., Camara, M., and Williams, P. (2007). Biosensor-based assays for PQS, HHQ and related 2-alkyl-4-quinolone quorum sensing signal molecules. *Nat Protoc* 2, 1254-1262. doi: 10.1038/nprot.2007.158.

Groitl, B., Dahl, J.U., Schroeder, J.W., and Jakob, U. (2017). *Pseudomonas aeruginosa* defense systems against microbicidal oxidants. *Mol Microbiol* 106, 335-350. doi: 10.1111/mmi.13768.

Kihara, A., Akiyama, Y., and Ito, K. (1996). A protease complex in the *Escherichia coli* plasma membrane: HflKC (HflA) forms a complex with FtsH (HflB), regulating its proteolytic activity against SecY. *Embo J* 15, 6122-6131.

Lee, C., Wigren, E., Trcek, J., Peters, V., Kim, J., Hasni, M.S., et al. (2015). A novel protein quality control mechanism contributes to heat shock resistance of worldwide-distributed *Pseudomonas aeruginosa* clone C strains. *Environ Microbiol* 17, 4511-4526. doi: 10.1111/1462-2920.12915.

Matuschek, E., Brown, D.F., and Kahlmeter, G. (2014). Development of the EUCAST disk diffusion antimicrobial susceptibility testing method and its implementation in routine microbiology laboratories. *Clin Microbiol Infect* 20, O255-266. doi: 10.1111/1469-0691.12373.

Morris, J.C. (1966). The acid ionization constant of HOCl from 5 to 35°. *J Phys Chem* 70, 3798-3805. doi: 10.1021/j100884a007.

O'Toole, G.A. (2011). Microtiter dish biofilm formation assay. *J Vis Exp* (47). doi: 10.3791/2437.

Pustelny, C., Brouwer, S., Musken, M., Bielecka, A., Dotsch, A., Nimtz, M., et al. (2013). The peptide chain release factor methyltransferase PrmC is essential for pathogenicity and environmental adaptation of Pseudomonas aeruginosa PA14. *Environ Microbiol* 15, 597-609. doi: 10.1111/1462-2920.12040.

Rashid, M.H., and Kornberg, A. (2000). Inorganic polyphosphate is needed for swimming, swarming, and twitching motilities of *Pseudomonas aeruginosa*. *Proc Natl Acad Sci* USA 97, 4885-4890. doi: 10.1073/pnas.060030097.

Shen, B., and Lutkenhaus, J. (2009). The conserved C-terminal tail of FtsZ is required for the septal localization and division inhibitory activity of MinC(C)/MinD. *Mol Microbiol* 72, 410-424.

Tavares, I.M., Leitão, J.H., and Sá-Correia, I. (2003). Chromosomal organization and transcription analysis of genes in the vicinity of *Pseudomonas aeruginosa glmM* gene encoding phosphoglucosamine mutase. *Biochem Biophy Res Commun* 302, 363-371. doi: http://dx.doi.org/10.1016/S0006-291X(03)00169-4.

Toska, J., Sun, Y., Carbonell, D.A., Foster, A.N., Jacobs, M.R., Pearlman, E., et al. (2014). Diversity of virulence phenotypes among type III secretion negative *Pseudomonas aeruginosa* clinical isolates. *PLoS One* 9, e86829. doi: 10.1371/journal.pone.0086829.
